# Venous thromboembolic and bleeding risk in population with obesity hospitalized for an acute medical illness receiving enoxaparin for thromboprophylaxis

**DOI:** 10.1016/j.rpth.2026.106628

**Published:** 2026-04-30

**Authors:** Walter Ageno, Juan I. Arcelus, Christine Stroh, Yasmina Djoudi, Mohamed Abdel-Moneim, Irfan Khan, Ekaterina Ponomareva, Marc Carrier

**Affiliations:** 1 Department of Medicine, University of Padua, Padua, Italy; 2 Department of Surgery, University of Granada Medical School and Hospital Universitario Virgen de las Nieves, Granada, Spain; 3 Department of Obesity and Metabolic Surgery and Proctology, SRH Hospital Gera, Gera, Germany; 4 Sanofi France, Paris, France; 5 Sanofi, UAE; 6 University of Sharjah, Sharjah, UAE; 7 Sanofi US, New Jersey, USA; 8 Axtria Inc, New Jersey, USA; 9 Ottawa Hospital Research Institute, Ottawa Hospital, University of Ottawa, Ottawa, Canada

**Keywords:** enoxaparin, hemorrhage, hospitalization, obesity, venous thromboembolism

## Abstract

**Background:**

Patients with obesity hospitalized for acute medical illness are at increased venous thromboembolism (VTE) risk, but evidence on predictors of VTE and major bleeding (MB) to optimize individual risk stratification and thromboprophylaxis remains limited, in particular for patients with extreme obesity.

**Objectives:**

This study assessed pharmacologic regimen, event rates, and predictors for VTE and MB among hospitalized medical patients with obesity receiving thromboprophylaxis using enoxaparin.

**Methods:**

Patients with body mass index (BMI) of ≥30 kg/m^2^ hospitalized for acute medical illness who received thromboprophylaxis with enoxaparin were selected from the Optum database. Event rates over a 90-day follow-up after enoxaparin initiation were estimated via the Kaplan–Meier method. Factors associated with outcome events were identified via Cox proportional hazard models.

**Results:**

Among 58,186 eligible patients, 17,398 (30%) had a BMI of >40 kg/m^2^; 56.9% received high-dose enoxaparin (>40 mg), and 42.8% received the standard dose (≤40 mg). The median duration of enoxaparin prophylaxis (3 days) was shorter than the length of the hospitalization (4 days). The 90-days cumulative incidence was 3.2% (95% CI, 3.1-3.4) for VTE and 1.8% (95% CI, 1.7-1.9) for MB. The highest VTE rates were observed in patients with cancer-related hospitalizations (7.8%). A history of VTE and MB were the strongest predictors of VTE (HR, 4.1; 95% CI, 3.6-4.7) and MB (HR, 2.8; 95% CI, 2.0-3.7), respectively.

**Conclusion:**

Acutely ill patients with obesity received enoxaparin for shorter duration than their hospitalization, with over half receiving a high-dose adjustment by weight. The VTE rates were nonnegligible in this population and exceed MB rates across illness subgroups, suggesting a need for individualized risk stratification to optimize thromboprophylaxis.

## Essentials

• Patients with obesity hospitalized with medical illness face high VTE and MB risks.
• Treatment patterns, nonnegligible VTE rates, and predictors were estimated using claims data.
• This population showed overall short prophylaxis duration and low use of weight-adjusted dosing.
• Patients with higher BMI and VTE rates had received higher doses of enoxaparin.

## Graphical abstract

**Figure:**
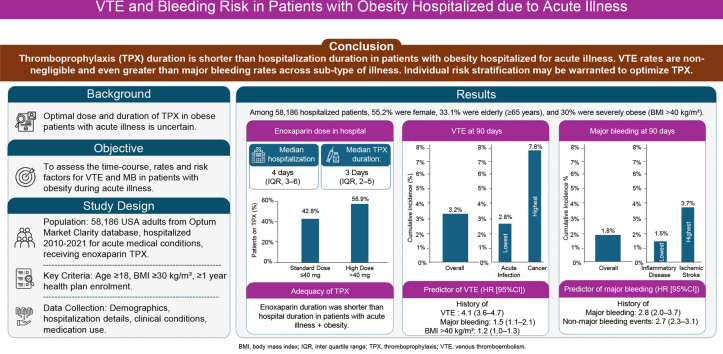

## 1 Introduction

Hospitalized patients with acute medical illnesses are at an increased risk of venous thromboembolism (VTE), including deep vein thrombosis (DVT) and pulmonary embolism (PE), as well as major bleeding (MB), an adverse event associated with thromboprophylaxis [1,2]. The risk of VTE in medically ill patients extends well beyond hospitalization, with about half of cumulative 6-month VTE events diagnosed within the first month despite prophylaxis, the majority of which (57%) occur after discharge [3]. Another study found that 66.9% of DVT/PE events were diagnosed within 30 days postdischarge [4]. However, pharmacologic prophylaxis is only being prescribed to a limited proportion of hospitalized patients, highlighting potentially suboptimal thromboprophylactic practices [5].

Several factors, including pre-existing conditions and the nature of the acute illness, contribute to the underlying risks of VTE [[6], [7], [8], [9]]. Obesity, defined as a body mass index (BMI) of ≥30 kg/m^2^, is a well-established independent risk factor for VTE in patients hospitalized with an acute medical illness [10,11]. The risk is estimated to be more than twice in patients with obesity than that in healthy weight individuals and increases when obesity interacts with additional thrombotic risk factors [12,13]. Given that obesity is affecting >1 billion people in 2024 and the incidence of obesity increased from 37.9% in 2017 and 2018 to 39.8% in 2021 among adult inpatients in the United States, hospitalized medical patients with obesity represent a substantial high-risk group for VTE, making thromboprophylaxis a critical concern in this patient population [14,15].

Despite the increased risk of VTE observed in patients with obesity hospitalized for acute medical illnesses, existing randomized controlled trials provide limited evidence regarding optimal thromboprophylactic strategies, particularly for patients with extreme obesity (BMI ≥ 40 kg/m^2^) [[16], [17], [18], [19], [20], [21]]. Optimal thromboprophylaxis entails risk stratification, selecting the appropriate anticoagulant type, determining the correct dose, and defining the suitable duration while minimizing the risk of bleeding complications. However, there is no consistency across practice guidelines and clinical practices regarding thromboprophylaxis among patients with obesity hospitalized for acute illness [22]. The 2018 American Society of Hematology (ASH) favor low-molecular-weight heparin (LMWH) over direct oral anticoagulant among acutely ill hospitalized medical patients with obesity; however, it lacks specific dosing and duration recommendations [16].

In this large database study, we aimed to describe pharmacologic regimen for thromboprophylaxis using enoxaparin in hospitalized medical patients with obesity. This study also assessed event rates and predictors for VTE and MB in this population.

## 2 Methods

### 2.1 Study population

This study used data from the Optum Market Clarity database and represents individuals in the United States enrolled in health plans including private and Medicare Advantage. The latter is a plan that contracts with Medicare (federal government health plan) to provide coverage to individuals mostly ≥65 years old. The Optum Market Clarity Database is a comprehensive health care claims resource that offers insights into patient demographics, hospital utilization, market trends, and provider performance. The database integrates linked information from electronic health records (EHRs), inpatient and outpatient claims, prescriptions, laboratory results, and plan enrolment [23].

Patients with index hospitalization for acute medical illness or surgery, between February 28, 2010, and June 30, 2021, and initiating thromboprophylaxis using enoxaparin were initially selected before the application of subsequent selection criteria. A single LMWH—enoxaparin—was selected as thromboprophylaxis in this study to facilitate the interpretation of findings within the context of a fixed intervention strategy, consistent with current guideline recommendations favoring LMWH over alternative agents in the setting of acute medical illness [16,[23], [24], [25]]. Based on previous trials comparing different anticoagulants for thromboprophylaxis in acutely ill medical patients [[26], [27], [28], [29]], the inclusion criteria comprised patients primarily hospitalized for acute medical illness representing the following conditions: infection, respiratory insufficiency, ischemic stroke, inflammatory condition, cancer, or heart failure. Stratification in this manner is purely descriptive, and all inferential analyses will treat the cohort as a single population with illness type as a covariate. International Classification of Diseases (ICD)-9 and ICD-10 diagnosis codes (Supplementary Table 1) in primary position, as noted in Optum claims and EHR, were used to determine the acute medical condition. Patients were allowed to have multiple acute illnesses as the reason for hospitalization. However, repeated episodes of the same acute illness were counted only once [30].

Additional inclusion criteria were age ≥18 years; ≥1 year of continuous enrollment in a health plan prior to index—defined as the start of enoxaparin thromboprophylaxis; and BMI of ≥30 kg/m^2^. Patients were excluded if they had VTE or an MB event 90 days prior to index, major surgery within 2 to 90 days prior to index, ongoing anticoagulant therapy (medication supply within 2 to 32 days prior to index), atrial fibrillation, chronic kidney disease stages IV and V (identified through diagnosis codes or estimated glomerular filtration rate < 30 mL/min/1.73m^2^), or dialysis. Finally, patients whose index hospitalization was for surgery were excluded. These surgical admissions were identified using a combination of Current Procedural Terminology codes and ICD-9 and ICD-10 procedure codes.

### 2.2 Data collection

Coexisting conditions were identified from medical diagnosis and procedure codes during the 1-year baseline period prior to index. Data on patient demography, index hospitalization, clinical conditions, and medication use were captured. Index hospitalization was characterized by length of stay, days since admission to enoxaparin start, and intensive care unit stay and patients with enoxaparin prescription postdischarge. Ongoing medication use was ascertained from medication supply within 90 days of index; a 30-day grace period was allowed after the end of days of supply.

### 2.3 Outcome measures

The duration of thromboprophylaxis represented the time from first enoxaparin administration to either the last administration in hospital or the last day of medication supply if the patient received postdischarge thromboprophylaxis. If there was an interruption of ≤2 days, the duration of thromboprophylaxis was assumed as continuous. Patients were categorized as receiving a standard dose if the daily dose administered at index was ≤40 mg, while those who received >40 mg were categorized as receiving a higher dose (Supplementary Figure 1).

The effectiveness outcome was VTE, defined as an acute event comprising DVT or PE, either fatal or nonfatal. VTE events were captured in both inpatient and outpatient settings, irrespective of whether they resulted in hospitalization. Cases of DVT or PE not leading to hospitalization were identified through medical record documentation of a newly diagnosed incident event. The safety outcome was MB, characterized as an acute event in the inpatient setting.

The ascertainment of VTE and MB end points was based on an algorithm that used diagnosis codes along with information on the setting (inpatient or outpatient). The algorithm, summarized in Supplementary Tables 2 and 3, was informed by prior studies and refined by coauthors to ensure specificity for identifying acute VTE and MB events. Patients were followed up for VTE and MB events for up to 90 days from index, with censoring criteria defined as death, disenrollment from the health plan, or the end of the 90-day follow-up period, whichever came first.

### 2.4 Statistical analysis

Baseline characteristics were reported descriptively for the overall cohort and by subgroups stratified by reason for hospitalization (Supplementary Table 4). Unadjusted means of baseline variables were also compared between high-dose and standard-dose enoxaparin groups using chi-squared test. Cumulative incidence of VTE and MB events were estimated via survival analysis (1 − Kaplan–Meier). Cox proportional hazards models adjusted for all baseline characteristics were used to investigate the predictors of VTE and MB. The full model approach ensures transparency and reduces the risk of overfitting, while maintaining all clinically relevant variables regardless of statistical significance.

## 3 Results

### 3.1 Baseline clinical conditions and medications

**Figure 1:**
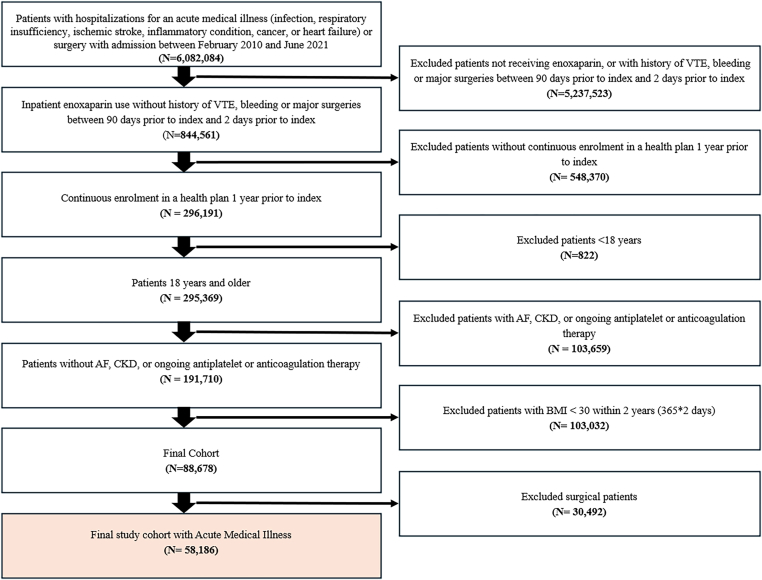
Selection of the study population. AF, atrial fibrillation; BMI, body mass index; CKD, chronic kidney disease; VTE, venous thromboembolism.

Overall, 58,186 patients met the selection criteria (Figure 1). The majority of patients were females, accounting for 55.2% of the study population. One-third (33.1%) of patients were aged 65 years and older, while 30% had a BMI of >40 kg/m^2^. Hypertension was the most prevalent comorbidity, affecting 54.3% of patients, followed by tobacco use in 47.5%. The most commonly used medications 90 days prior to index hospitalization were angiotensin-converting enzyme inhibitors/angiotensin II receptor blockers (31.2%), followed by statins (26.1%). Summarized in Table 1 are the baseline characteristics for the patients.

**Table 1:** Baseline characteristics for patients with acute medical illness.

Characteristic	Value (*N* = 58,186)
Reason for hospitalization	
Heart failure only	7.8
Infection	56.5
Respiratory insufficiency	30.7
Ischemic stroke	8.3
Inflammatory condition	8.2
Cancer	8.1
Demographics	
Female sex (%)	55.2
Age (y) (%)	
18-39	11.6
40-64	55.3
65-75	20.6
75	12.5
BMI (kg/m^2^)	
30-34.9	44.7
35-39.9	25.3
>40	29.9
Index hospitalization characteristics (at follow-up)	
Length of hospital stay (d)	5 (3-7)
Admission to enoxaparin start (d)	1 (0-1)
Enoxaparin start to discharge (d)	4 (3-6)
Duration of prophylaxis with enoxaparin	3 (2-5)
Patients with enoxaparin Rx postdischarge	1.3
Stroke and CBVD	
Hemorrhagic stroke	0.4
Ischemic stroke	5.2
Unspecified stroke or CBVD without stroke	8.7
Other comorbidities	
Thrombophilia	0.5
Severe varicosities	2.1
History of cancer	14.3
Gastroduodenal ulcer	5.0
Lower limb paralysis	0.7
Central venous catheter	4.6
Heart failure	10.9
COPD	21.0
CKD stage III	18.4
CHD	17.9
Drug misuse disorder^a^	23.2
HIV infection	1.4
Current or former of tobacco use	47.5
Hypertension	54.3
Diabetes	34.2
Anemia	13.3
Immunohematologic conditions^b^	2.0
Peripheral vascular disease	9.3
Moderate/severe chronic liver disease	6.3
History of VTE and bleeding	
History of VTE	4.5
History of bleeding	
Major bleeding	1.3
Nonmajor bleeding	10.0
Medication use	
Anticoagulants^c^	24.9
ACEi/ARB	31.2
β-Blockers	14.7
CCBs	16.0
Statins	26.1
Antiplatelets^c^	4.1
Hormone replacement therapy	0.7

Values are given as % or median (IQR).

ACEi, angiotensin-converting enzyme inhibitor; ARB, angiotensin II receptor blocker; CBVD, cerebrovascular disease; CCB, calcium channel blocker; CHD, coronary heart disease; CKD, chronic kidney disease; COPD, chronic obstructive pulmonary disease; PAD, peripheral artery disease; VTE, venous thromboembolism.

a Includes codes for nicotine dependence, opioid dependence, other psychoactive substance abuse, and other illegal prescription drug abuse.

b Includes anemia, cell aplasia, pancytopenia, bone marrow failure syndromes, agranulocytosis, genetic anomalies of leukocytes, severe combined immunodeficiency, Nezelof syndrome, Wiskott-Aldrich syndrome, Di George syndrome, and acute graft-vs-host disease.

c Within 30-90 days of index.

### 3.2 Pharmacologic regimen

#### 3.2.1 Thromboprophylaxis dose

A total of 33,124 patients (56.9%) received a high dose of enoxaparin, while 24,889 patients (42.8%) received a standard dose. The average daily high dose of enoxaparin was 67 mg. The highest average dose (73.35 mg) of enoxaparin was observed in patients admitted for heart failure (Supplementary Table 5).

The distribution of patients receiving high-dose vs standard-dose enoxaparin varied significantly across age groups and BMI categories (Table 2), with the most notable difference observed in the 65- to 75-year age group (21.2% vs 19.7%, respectively) and 40-kg/m^2^ BMI group (33.2% vs 25.6%, respectively). Patients who were statistically more likely to receive high- vs standard-dose enoxaparin included those with unspecified stroke or cerebrovascular disease without stroke (9.7% vs 7.5%), a history of cancer (14.9% vs 13.4%), lower limb paralysis (0.8% vs 0.5%), central venous catheter (5.2% vs 3.7%), heart failure (11.8% vs 9.7%), coronary artery disease (18.6% vs 16.9%), hypertension (55.2% vs 53%), and diabetes (34.9 vs 33.3%) (Table 2).

**Table 2:** Pharmacologic dosage for patients with acute medical illness.

Characteristic	Enoxaparin dose^a^			
	Standard dose (≤40 mg/d), *N* = 24,889		High dose (>40 mg/d), *N* = 33,124	
Demographics				
Female sex	13,664	54.9	18,351	55.4
Male sex	11,225	45.1	14,773	44.6
Age (y)				
18-39	3136	12.6	3644	11
40-64	13,838	55.6	18,251	55.1
65-75	4903	19.7	7022	21.2
>75	3036	12.2	4207	12.7
BMI (kg/m^2^)				
30-34.9	12,096	48.6	13,813	41.7
35-39.9	6421	25.8	8314	25.1
>40	6372	25.6	10,997	33.2
Index hospitalization characteristics (at follow-up)				
Length of hospital stay (d)	5	3-7	5	3-8
Admission to enoxaparin start (d)	1	0-1	1	0-1
Enoxaparin start to discharge (d)	4	3-6	4	3-7
Duration of prophylaxis with enoxaparin	3	2-5	3	2-6
Patients with enoxaparin Rx postdischarge	224	0.9	530	1.6
Stroke and CBVD				
Hemorrhagic stroke	100	0.4	132	0.4
Ischemic stroke	1244	5	1756	5.3
Unspecified stroke or CBVD without stroke	1867	7.5	3213	9.7
Other comorbidities				
Thrombophilia	100	0.4	199	0.6
Severe varicosities	498	2	696	2.1
History of cancer	3335	13.4	4935	14.9
Gastroduodenal ulcer	1244	5	1656	5
Lower limb paralysis	124	0.5	265	0.8
Central venous catheter	921	3.7	1722	5.2
Heart failure	2414	9.7	3909	11.8
COPD	5177	20.8	7022	21.2
CKD stage III	4754	19.1	5896	17.8
CHD	4206	16.9	6161	18.6
Drug misuse disorder^b^	5899	23.7	7552	22.8
HIV infection	398	1.6	397	1.2
Current or former of tobacco use	11,748	47.2	14,906	45
Hypertension	13,191	53	18,284	55.2
Diabetes	8288	33.3	11,560	34.9
Anemia	3236	13	4472	13.5
Immunohematologic conditions^c^	498	2	696	2.1
Peripheral vascular disease	2389	9.6	3014	9.1
History of VTE and bleeding				
Moderate/severe chronic liver disease	1618	6.5	2021	6.1
History of VTE	946	3.8	1623	4.9
Major bleeding	324	1.3	397	1.2
Nonmajor bleeding	2439	9.8	3379	10.2
Medication use^d^				
Anticoagulants^e^	3360	13.5	11,063	33.4
ACEi/ARB	7716	31	10,401	31.4
β-Blockers	3609	14.5	4902	14.8
CCBs	4132	16.6	5167	15.6
Statins	5899	23.7	9208	27.8
Antiplatelets^e^	1020	4.1	1325	4
Hormone replacement therapy	174	0.7	232	0.7

Values are given as % or median (IQR).

ACEi, angiotensin-converting enzyme inhibitors; ARB, angiotensin II receptor blocker; CBVD, cerebrovascular disease; CCB, calcium channel blocker; CHD, coronary heart disease; CKD, chronic kidney disease; COPD, chronic obstructive pulmonary disease; PAD, peripheral artery disease; VTE, venous thromboembolism.

a Of 58,186 patients, 173 in the acute medical illness group had missing dosage.

b Includes codes for nicotine dependence, opioid dependence, other psychoactive substance abuse, and other illegal prescription drug abuse.

c Includes anemia, cell aplasia, pancytopenia, bone marrow failure syndromes, agranulocytosis, genetic anomalies of leukocytes, severe combined immunodeficiency, Nezelof syndrome, Wiskott-Aldrich syndrome, Di George syndrome, and acute graft-vs-host disease.

d Within 3 months prior to index.

e Within 30-90 days of index.

#### 3.2.2 Thromboprophylaxis duration

**Figure 2:**
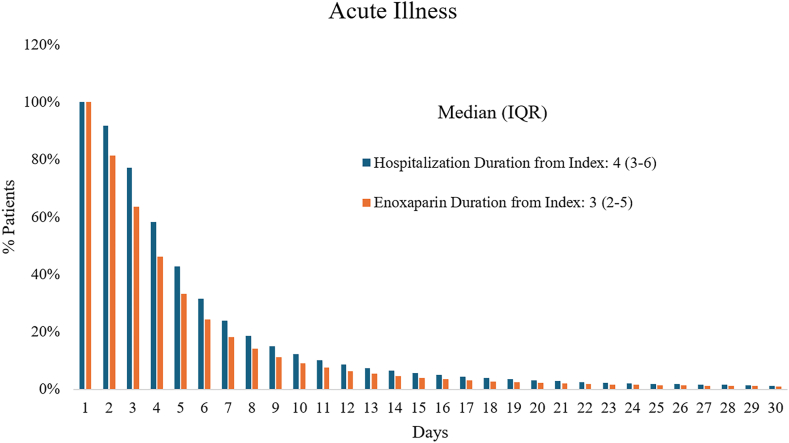
Duration of hospitalization and enoxaparin.

The median duration of hospitalization was 4 days (IQR, 3-6 days). Enoxaparin was discontinued prior to discharge with the median duration of enoxaparin prophylaxis of 3 days (IQR, 2-5 days). Of the total cohort, 18% of patients were on enoxaparin for 7 days or more, and only 1% of the patients were receiving enoxaparin at 30 days (Figure 2). While median duration of prophylaxis was the same for those treated with high and standard doses, almost twice as many patients receiving high-dose enoxaparin filled enoxaparin prescriptions postdischarge (1.6% vs 0.9%).

### 3.3 Event rates and predictors for VTE and MB

**Figure 3:**
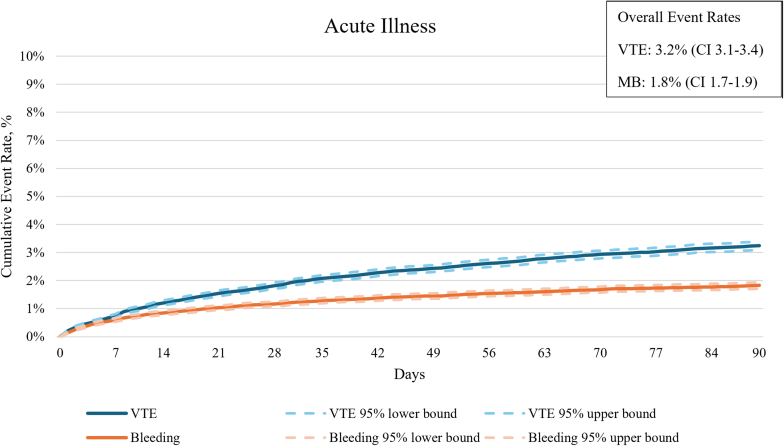
Venous thromboembolism (VTE) and major bleeding (MB) event rates for acute illness.

**Figure 4:**
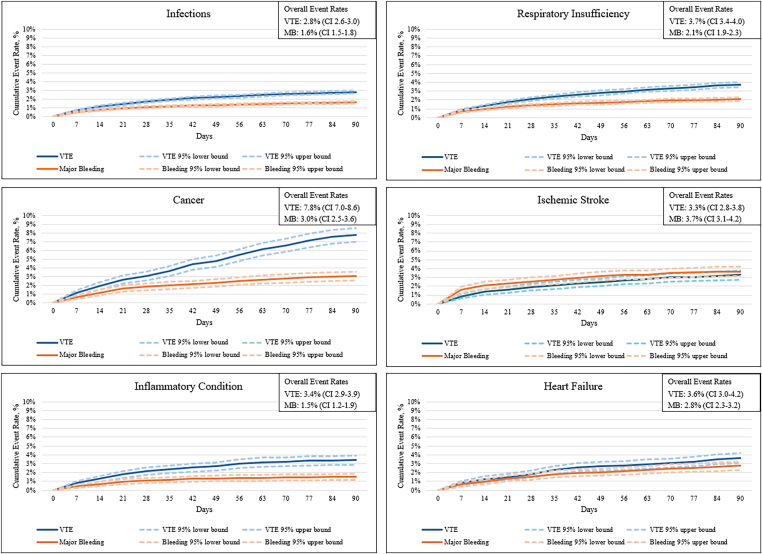
Venous thromboembolism (VTE) and major bleeding (MB) rates for select index hospitalizations in the acute illness group. Time zero indicates enoxaparin initiation. KM, Kaplan–Meier; VTE, venous thromboembolism.

The event rates reported by reason for hospitalization are descriptively summarized in Table 3. The cumulative VTE event rates were 0.7% (95% CI, 0.7-0.8), 1.9% (95% CI, 1.8-2.0), and 3.2% (95% CI, 3.1-3.4) at the 7-, 30-, and 90-day follow-ups, and the MB event rates were 0.6% (95% CI, 0.5-0.7), 1.2% (95% CI, 1.1-1.3), and 1.8% (95% CI, 1.7-1.9) (Figure 3, Table 3), respectively. Patients with cancer-related hospitalizations (7.8%) had the highest cumulative incidence of VTE at the 90 days follow-up, while those hospitalized with acute infections (2.8%) had the lowest; the highest and lowest MB event rates at 90 days were observed in ischemic stroke (3.7%) and inflammatory disease (1.5%), respectively (Figure 4).

**Table 3:** Event rates for VTE and major bleeding.

Reason for hospitalization	*n* (%)	VTE						Major bleeding					
		7 d		30 d		90 d		7 d		30 d		90 d	
		Cumulative outcome events (*n*)	% (CI)	Cumulative outcome events (*n*)	% (CI)	Cumulative outcome events (*n*)	% (CI)	Cumulative outcome events (*n*)	% (CI)	Cumulative outcome events (*n*)	% (CI)	Cumulative outcome events (*n*)	% (CI)
Acute illness overall	58,186	423	0.7 (0.7-0.8)	1061	1.9 (1.8-2.0)	1795	3.2 (3.1-3.4)	343	0.6 (0.5-0.7)	686	1.2 (1.1-1.3)	1030	1.8 (1.7-1.9)
Infection	32,893 (56.5)	229	0.7 (0.6-0.8)	564	1.8 (1.6-1.9)	878	2.8 (2.6-3.0)	174	0.5 (0.5-0.6)	354	1.1 (1.0-1.2)	518	1.6 (1.5-1.7)
Respiratory insufficiency	17,918 (30.8)	146	0.8 (0.7-1.0)	369	2.2 (1.9-2.4)	624	3.7 (3.5-4.0)	122	0.7 (0.6-0.8)	252	1.4 (1.3-1.6)	359	2.1 (1.9-2.3)
Cancer	4688 (8.1)	51	1.1 (0.8-1.4)	144	3.2 (2.7-3.7)	328	7.8 (7.0-8.6)	30	0.6 (0.4-0.9)	86	1.9 (1.5-2.3)	134	3.0 (2.5-3.6)
Ischemic stroke	4881 (8.3)	41	0.8 (0.6-1.1)	96	2.0 (1.6-2.4)	154	3.3 (2.8-3.8)	76	1.6 (1.2-1.9)	125	2.6 (2.2-3.1)	173	3.7 (3.1-4.2)
Inflammatory condition	4774 (8.2)	38	0.8 (0.6-1.1)	103	2.2 (1.8-2.6)	157	3.4 (2.9-3.9)	21	0.4 (0.3-0.6)	53	1.1 (0.8-1.4)	71	1.5 (1.2-1.9)
Heart failure	4596 (7.9)	35	0.7 (0.5-1.0)	86	1.9 (1.5-2.4)	155	3.6 (3.0-4.2)	30	0.7 (0.4-0.9)	75	1.7 (1.3-2.0)	122	2.8 (2.3-3.2)

Event rates represent the 1 – Kaplan–Meier estimates. Adjusted number of events indicate the event rate multiplied by *N*.

VTE, venous thromboembolism.

Results from multivariable analysis for associations between baseline characteristics and outcomes are shown in Table 4. Patients with cancer-related hospitalizations had 2 times greater risk of VTE (hazards ratio [HR], 2.6; 95% CI, 2.2-3.1), while patients hospitalized due to ischemic stroke had almost a 3-fold greater risk of MB (HR, 2.8; 95% CI, 2.3-3.5), compared with patients hospitalized for heart failure alone.

**Table 4:** Patient characteristics at index associated with the occurrence of VTE and major bleeding during 1 to 90 days postindex: Cox proportional hazards model.^a^

Characteristic	*n (%)*	VTE adjusted HR (95% CI)	MB adjusted HR (95% CI)
Reason for hospitalization			
Heart failure only	4596 (7.8)	Ref	Ref
Infection	32,893 (56.5)	1.1 (1.0-1.3)	1.3 (1.1-1.5)
Respiratory insufficiency	17,918 (30.7)	1.5 (1.3-1.6)	1.6 (1.4-1.9)
Ischemic stroke	4881 (8.3)	1.3 (1.1-1.6)	2.8 (2.3-3.5)
Inflammatory condition	4774 (8.2)	1.3 (1.1-1.6)	1.2 (0.9-1.5)
Cancer	4688 (8.1)	2.6 (2.2-3.1)	2.3 (1.8-3.0)
BMI (kg/m^2^)			
30-34.9	26,016 (44.7)	Ref	Ref
35-39.9	14,745 (25.3)	1.0 (0.9-1.1)	0.9 (0.8-1.1)
>40	17,425 (29.9)	1.2 (1.0-1.3)	0.9 (0.8-1.1)
Stroke and CBVD			
No stroke or CBVD	50,075 (86.1)	Ref	Ref
Ischemic stroke	3034 (5.2)	1.2 (1.0-1.5)	1.2 (1.0-1.5)
Unspecified stroke or CBVD without stroke	5077 (8.7)	1.0 (0.9-1.2)	1.1 (0.9-1.3)
Other comorbidities			
Thrombophilia	1149 (0.5)	1.9 (1.3-2.6)	1.8 (1.0-3.2)
Severe varicosities	1206 (2.1)	1.1 (0.8-1.5)	0.9 (0.6-1.4)
History of cancer	31,193 (13.4)	1.2 (1.1-1.4)	0.9 (0.7-1.1)
Gastroduodenal ulcer	2936 (5.0)	1.2 (1.0-1.4)	1.1 (0.9-1.4)
Lower limb paralysis	393 (0.7)	1.6 (1.0-2.4)	1.2 (0.7-2.3)
Central venous catheter	2,663 (4.6)	1.4 (1.2-1.6)	1.2 (0.9-1.5)
Heart failure	6360 (10.9)	1.0 (0.8-1.1)	1.1 (0.9-1.3)
COPD	12,228 (21.0)	1.1 (0.9-1.2)	0.8 (0.7-1.0)
CKD stage III	10,687 (18.4)	1.1 (1.0-1.3)	1.3 (1.1-1.5)
CHD	10,404 (17.9)	0.8 (0.7-0.9)	1.1 (0.9-1.3)
Drug misuse disorder	13,489 (23.2)	1.1 (0.9-1.2)	1.1 (0.9-1.3)
HIV infection	794 (1.4)	0.8 (0.6-1.3)	1.4 (0.9-2.3)
Current or former of tobacco use	14,972 (49.1)	1.0 (0.9-1.1)	1.0 (0.8-1.1)
Hypertension	31,569 (54.3)	1.0 (0.9-1.1)	1.0 (0.9-1.2)
Diabetes	19,906 (34.2)	1.0 (0.9-1.1)	1.2 (1.0-1.4)
Anemia	7737 (13.3)	1.2 (1.0-1.3)	1.2 (1.0-1.4)
Immunohematologic conditions	1175 (2.0)	1.1 (0.9-1.5)	1.3 (0.9-1.8)
Peripheral vascular disease	5423 (9.3)	1.0 (0.9-1.2)	1.0 (0.8-1.2)
Moderate/severe chronic liver disease	3675 (6.3)	0.9 (0.7-1)	1.2 (1.0-1.5)
History of VTE	2603 (4.5)	4.1 (3.6-4.7)	1.2 (1.0-1.6)
History of major bleeding	730 (1.3)	1.5 (1.1-2.1)	2.8 (2.0-3.7)
History of nonmajor bleeding	5829 (10.0)	0.9 (0.8-1.0)	2.7 (2.3-3.1)
Medication use			
Anticoagulants	14,490 (24.9)	1.1 (0.9-1.2)	0.9 (0.8-1.1)
ACEi/ARB	18,177 (31.2)	0.9 (0.8-1.0)	1.2 (1.0-1.3)
β-Blockers	8570 (14.7)	1.0 (0.9-1.2)	1.1 (0.9-1.3)
CCBs	9325 (16.0)	1.0 (0.9-1.2)	0.9 (0.8-1.1)
Statins	15,162 (26.1)	1.0 (0.9-1.1)	1.0 (0.9-1.1)
Antiplatelets	2358 (4.1)	1.0 (0.8-1.3)	1.3 (1.0-1.7)
Hormone replacement therapy	426 (0.7)	1.2 (0-2.2)	1.4 (0.7-3)

HRs and CIs are rounded to 1 decimal place.

ACEi, angiotensin-converting enzyme inhibitor; ARB, angiotensin II receptor blocker; CBVD, cerebrovascular disease; CKD, chronic kidney disease; COPD, chronic obstructive pulmonary disease; HR, hazard ratio; MB, major bleeding; VTE, venous thromboembolism.

a All baseline characteristics were included in both models regardless of statistical significance.

The risk of VTE was nonsignificantly higher in patients with a BMI of >40 kg/m^2^ (HR, 1.2; 95% CI, 1.0-1.3) than in those in the 30- to 39.9-kg/m^2^ BMI groups. A history of VTE (HR, 4.1; 95% CI, 3.6-4.7) was a significant predictor for VTE but not a statistically significant predictor (HR, 1.2; 95% CI, 1.0-1.6) for MB. Patients with a history of non-MB events had a significantly higher risk for MB (HR, 2.7; 95% CI, 2.3-3.1). Patients with a history of MB had a significantly higher risk for both VTE (HR, 1.5; 95% CI, 1.1-2.1) and MB (HR, 2.8; 95% CI, 2.0-3.7).

## 4 Discussion

Our study showed that among 58,186 acutely ill patients with obesity included in the study, 56.9% of patients received a high dose of enoxaparin, while 42.8% received a standard dose. Furthermore, only 1.3% of the total study participants received a postdischarge enoxaparin prescription. The median duration of enoxaparin prophylaxis in this cohort of acutely ill patients with obesity was 3 days (IQR, 2-5 days), which was shorter than the length of the inpatient stay.

The proportion of patients with obesity hospitalized with acute illness receiving an enoxaparin prescription postdischarge was lower than the proportion observed in patients without obesity hospitalized with acute illness [2]. However, the median duration of enoxaparin prophylaxis in these acutely ill patients with obesity population was comparable with that of patients without obesity and with acute illness [2].

The study also reported a nonnegligible 90-day event rate of VTE and MB complications in patients with obesity hospitalized for acute medical illness (3.2% [95% CI, 3.1-3.4] and 1.8% [95% CI, 1.7-1.9] respectively). These findings are consistent with another large study among patients with obesity receiving enoxaparin in usual clinical practice, which reported high rates of both VTE and MB, which were the highest following hospital discharge (Supplementary Table 6) [11]. The highest 90-day event rate of VTE were those with a cancer-related hospitalization (7.8%). The absolute difference in the incidence rate for VTE and MB at 90 days was the highest for patients with obesity and cancer-related hospitalizations (absolute risk difference of 4.8%), suggesting a compelling case of considering thromboprophylaxis in this patient population. One avenue for further research in this area could be an analysis providing comprehensive risk estimates across all baseline predictors to provide a comprehensive overview.

We found significantly higher risk of VTE among patients older than 75 years than that in patients in the 18- to 39-year age group, as well as among males. A significantly increased risk of VTE was also observed in patients with a BMI of >40 kg/m^2^. These study findings are consistent with other recently conducted studies among patients in usual clinical practice settings. Several studies have previously investigated the association between BMI and the risk of VTE [22,31,32]. Similarly, previous studies identified that older age is an independent risk factor for VTE, which could likely be due to a higher incidence of comorbidities, which is associated with age [26,28]. Finally, a previous study of patients hospitalized with acute medical illness has also reported a greater risk for MB among male patients than that in female patients during a 180-day follow-up period [33].

Patients with a BMI of ≥40 kg/m^2^ were found to have the highest risk of VTE as an adverse event, although the association between a BMI of ≥40 kg/m^2^ and MB was not statistically significant. Additionally, this study observed that a significantly larger proportion of patients in the BMI ≥ 40 kg/m^2^ group received high-dose enoxaparin than those in other BMI groups. Together, these findings suggest a trend in enoxaparin prophylaxis associated with increasing BMI and VTE risk, which warrants further investigation to compare use of different doses of enoxaparin [[34], [35], [36]]. It has also been previously shown that a higher dose of thromboprophylaxis resulted in a higher reduction of VTE risk and no significant impact on MB, as compared with standard-dose thromboprophylaxis in patients with obesity (Supplementary Figure 2) [[33], [34], [35]].

Our study identified a clear pattern: the subgroup patients with a history of VTE faced a significantly higher risk of VTE yet were not at an increased risk of MB. Despite this elevated VTE risk, these patients were not more likely to receive high-dose enoxaparin or longer duration of thromboprophylaxis. The increasing cumulative VTE risk for the overall population seen in Figure 3 through day 90 could also be related to discontinuation of enoxaparin and/or confounding by indication in the dose and duration of thromboprophylaxis. These findings suggest a potential inconsistency in thromboprophylactic practices. Therefore, it underscores the importance of individualized risk assessment to balance the benefits of thromboprophylaxis against the potential for bleeding complications. However, current clinical practice guidelines do not specifically address optimal pharmacologic thromboprophylaxis regimens in patients with obesity hospitalized with medical illness. The 2018 ASH clinical practice guidelines do not offer specific recommendations on LMWH dose selection or the duration of thromboprophylaxis in this high-risk population [34]. Similarly, the American College of Chest Physicians Evidence-Based Clinical Practice Guidelines simply recommend thromboprophylaxis for acutely ill patients based on the level of thrombosis risk, patients’ preference, ease of administration, and cost [25].

Findings from existing literature, combined with our study results and current clinical practice guidelines recommendations, underscore the knowledge gap on optimal pharmacologic thromboprophylaxis in patients with obesity hospitalized for medical illness. Moreover, this study's findings highlight potential predictors that could help clinicians identify a particularly high-risk of complications. Risk-assessment models that provide reliable stratification of hospitalized patients with obesity according to their underlying risk of VTE or MB would help clinicians assessing the risks and benefits of pharmacologic thromboprophylaxis [20]. Hence, a careful evaluation is warranted on whether prevalent practice patterns and recommended strategies are optimal for the reduction in the risk of VTE.

Key strengths of our study include a large cohort (*N* = 58,186) and contemporary (2010-2021) population with obesity receiving pharmacologic thromboprophylaxis with enoxaparin. To our knowledge, this is one of the largest studies evaluating predictors for VTE and MB among patients with obesity hospitalized with medical illness receiving pharmacologic thromboprophylaxis. The population was well classified in terms of clinical conditions and medications at baseline as the different databases (EHR, claims, enrollment information, pharmacy, and laboratory data) were linked. Linked database analysis also offered high numbers of patients and covariates that increased the detection of VTE and MB following discharge from the hospital. An extended and consistent follow-up period of 90 days was also taken to monitor VTE and MB, which evaluated the long-term postdischarge VTE and MB risk in patients with obesity. We meticulously calibrated and enhanced the algorithm for the identification of VTE and MB over the methods used in other observational studies.

As this study was based on observational data, several limitations should be noted. First, it included only individuals in the United States who were enrolled under a commercial or Medicare Advantage health plan, which may not be an accurate representation of noninsured populations, those covered under a public insurance program other than Medicare Advantage (eg, Medicaid and other Medicare plans), or other international populations. Second, in contrast to the highly stringent criteria used in randomized controlled trials, the current analysis relied on an algorithm based on codes and other information available in the database to identify VTE and MB end points. Therefore, misclassification cannot be excluded. Third, there is a potential for selection bias in the study as enoxaparin was administered to all participants, and the decision to prescribe it may have been influenced by clinicians’ assessments of patients’ risk profiles. Fourth, MB events were identified only in the inpatient setting, which may result in differences in the severity profile of MB events compared with VTE events included in the study. Fifth, as death was not included as a competing event due to limited availability of mortality data, the cumulative incidence of VTE and MB may have been overestimated. Finally, the predictors in our study reflect patients’ status at index before follow-up; however, as the patient population represents hospitalized individuals, clinical characterization and predictors could have changed over the course of hospitalization, which were not accounted for in this study.

## 5 Conclusions

In this large population representing usual clinical practice setting with obesity hospitalized for acute medical illness, we found that, despite the use of enoxaparin thromboprophylaxis, the 90-day risk of VTE and MB remains considerable, particularly among patients with cancer-related hospitalizations and additional predictors such as advanced age, male sex, higher BMI, and a history of VTE or bleeding. Our findings highlight the complexity of balancing VTE prevention with MB risk in this high-risk population and emphasize the need for individualized risk assessment and dosing strategies. Given the current gaps in guideline recommendations, further prospective studies are warranted to optimize pharmacologic thromboprophylaxis strategies tailored to patients with obesity.

## Supplementary material

Supplementary Tables S1-S6 and Figures F1 and F2
