# Supplementary material for: Venous thromboembolic and bleeding risk in population with obesity hospitalized for an acute medical illness receiving enoxaparin for thromboprophylaxis
Source: Res Pract Thromb Haemost. 2026 Apr 30;10(4):106628. doi: 10.1016/j.rpth.2026.106628 (PMC13272523; doi:10.1016/j.rpth.2026.106628)
Supplement: Supplementary Tables S1-S6 and Figures F1 and F2 [file mmc1.docx]

# **Supplement**

## **Identification of the Acute Illness Cohort**

**Table S1. Codes for identification of the acute illness cohort (summarized)**

| **Criterion** | **Codes (ICD-9)** | **Codes (ICD-10)** |
| --- | --- | --- |
| Hospitalization with a primary diagnosis of: |  |  |
| Acute heart failure | 428 | I50 |
| Acute ischemic stroke | 433, 434 | I63 |
| Acute respiratory insufficiency | 278  466, 490-496,  500-508, 511, 512, 514-519 | E66  J20, J21, J40-J45, J47, J60-J70, J80-J82, J84, J86, J90-J96, J98, J99 |
| Acute infection | 003, 008, 010, 016, 022, 033, 035, 036, 038, 040, 041, 047, 049, 053, 054, 056, 062-064, 066, 072, 079, 091, 094, 098-99  100, 130, 131, 137  277  306, 320-323, 376  466, 480-488  510, 513, 538, 567, 569, 590  601, 646, 681, 682  711, 713, 728, 730, 747, 753, 785, 788, 790, 793  939, 995, 996  V02, V09 | A00-A09, A15, A17-A28, A30-A44, A46, A48-A60, A63-A71, A74, A75, A77-A96, A98, A99    B00-B10, B15-B20, B25-B27, B30, B33-B60, B64-B83, B85-B89, B95-B97, B99  G00-G07  H00, H10, H66, H70  J00-J06, J09-J18, J20-J22, J36, J37, J39, J85  K12, K57, K61, K63, K65, K75  L02, L05, L08, L30  M00, M01, M60, M86  N10, N15, N30, N34, N39, N41, N45, N48, N70, N73 N75, N99  O03, O04, O07, O08, O86  P23  R65  T82-T85  U07  Y84 |
| Acute inflammatory diseases | 359  556  607  614-616  714, 716, 720-724, 729  995 | B42  E24  G56, G61, G72, G98  K50, K51  L40  L93  M02, M04-M07, M10-M13, M15-M19, M1A, M25, M26, M31, M32, M35, M45, M46, M65, M70, M71, M77, M79, M85, M94  N48, N49, N71, N72, N76, N77  R65, R79 |
| Acute cancer (all sites excluding non-melanoma skin cancer) | 140-165, 170-176, 179-209 | C00-C26, C30-C34, C38-C41, C43, C45-C49, C4A, C50-C58, C60-C79, C7A, C7B, C80-C86, C88, C90-96 |

## **Identification of the VTE Endpoint**

The VTE endpoint was intended to represent a new onset event. It was identified by utilizing an algorithm based on the international classification of diseases 9^th^ revision (ICD-9) and 10^th^ revision (ICD-10) codes. Many observational studies have applied these criteria for VTE endpoint identification. [1-4] Validation of codes for incident VTE events have been undertaken in two relatively rigorous studies. The first one by White et al. 2010[5] was based on ICD-9 codes in a US population, and the second one by Sundbøll et al. 2016[6] was based on ICD-8^th^ revision (ICD-8) and ICD-10 codes in a Danish population. Of note, no validation study for incident VTE events focusing exclusively on ICD-10 codes has been undertaken to date. A key advantage of ICD-10 codes is substantially higher granularity for VTE, potentially translating to higher specificity and PPV.

The study by White et al. 2010[5] reported a PPV of 95% and 75% for incident VTE events for ICD-9 codes in inpatient primary and secondary positions, respectively. The study noted that the lower PPV of codes in secondary position was mainly due to the codes that are non-specific for thrombosis in a deep vein. The study by Sundbøll et al. 2016[6] reported a PPV of 91% in inpatient setting when the codes for VTE were used together with relevant imaging examination (ultrasound or CT scan) performed within a ± 10-day period. The PPV was 71% for codes in outpatient setting, however, it was not reported by how much this can be increased by also requiring relevant imaging examination (for inpatient setting, there was a relative increase of 18% for PPV with this requirement). For both inpatient and outpatient setting it was also not clear by how much the PPV can be improved by either removing non-specific codes, or strictly limiting codes to those that explicitly mention acute VTE event.

Considering these points, we developed the algorithm summarized in *Table S2*. We first reviewed and included all ICD-9 and ICD-10 codes that are specific to acute pulmonary embolism or deep vein thrombosis (DVT). We then removed all DVT codes that did not explicitly mention deep veins or identified veins that are deep (e.g., femoral). Next, we also removed codes that explicitly mentioned chronic. This set was used for identification of VTE events in inpatient setting. From this set, we then retained only those codes that explicitly mentioned acute onset event or pulmonary embolism in the definition and used these codes in outpatient setting.

**Table S2. Codes for identification of the VTE endpoint**

| **Code Type** | **Code** | **Description** | **Inpatient Primary** | **Inpatient Non-Primary** | **Outpatient** |
| --- | --- | --- | --- | --- | --- |
| ICD10 | I26 | Pulmonary embolism | X | X | X |
| ICD10 | I26.0 | Pulmonary embolism with acute cor pulmonale | X | X | X |
| ICD10 | I26.02 | Saddle embolus of pulmonary artery with acute cor pulmonale | X | X | X |
| ICD10 | I26.09 | Other pulmonary embolism with acute cor pulmonale | X | X | X |
| ICD10 | I26.9 | Pulmonary embolism without acute cor pulmonale | X | X | X |
| ICD10 | I26.92 | Saddle embolus of pulmonary artery without acute cor pulmonale | X | X | X |
| ICD10 | I26.93 | Single subsegmental pulmonary embolism without acute cor pulmonale | X | X | X |
| ICD10 | I26.94 | Multiple subsegmental pulmonary emboli without acute cor pulmonale | X | X | X |
| ICD10 | I26.99 | Other pulmonary embolism without acute cor pulmonale | X | X | X |
| ICD10 | I80.1 | Phlebitis and thrombophlebitis of femoral vein | X | X | — |
| ICD10 | I80.10 | Phlebitis and thrombophlebitis of unspecified femoral vein | X | X | — |
| ICD10 | I80.11 | Phlebitis and thrombophlebitis of right femoral vein | X | X | — |
| ICD10 | I80.12 | Phlebitis and thrombophlebitis of left femoral vein | X | X | — |
| ICD10 | I80.13 | Phlebitis and thrombophlebitis of femoral vein, bilateral | X | X | — |
| ICD10 | I80.2 | Phlebitis and thrombophlebitis of other and unspecified deep vessels of lower extremities | X | X | — |
| ICD10 | I80.20 | Phlebitis and thrombophlebitis of unspecified deep vessels of lower extremities | X | X | — |
| ICD10 | I80.201 | Phlebitis and thrombophlebitis of unspecified deep vessels of right lower extremity | X | X | — |
| ICD10 | I80.202 | Phlebitis and thrombophlebitis of unspecified deep vessels of left lower extremity | X | X | — |
| ICD10 | I80.203 | Phlebitis and thrombophlebitis of unspecified deep vessels of lower extremities, bilateral | X | X | — |
| ICD10 | I80.209 | Phlebitis and thrombophlebitis of unspecified deep vessels of unspecified lower extremity | X | X | — |
| ICD10 | I80.21 | Phlebitis and thrombophlebitis of unspecified deep vessels of unspecified lower extremity | X | X | — |
| ICD10 | I80.211 | Phlebitis and thrombophlebitis of right iliac vein | X | X | — |
| ICD10 | I80.212 | Phlebitis and thrombophlebitis of left iliac vein | X | X | — |
| ICD10 | I80.213 | Phlebitis and thrombophlebitis of iliac vein, bilateral | X | X | — |
| ICD10 | I80.219 | Phlebitis and thrombophlebitis of unspecified iliac vein | X | X | — |
| ICD10 | I80.22 | Phlebitis and thrombophlebitis of popliteal vein | X | X | — |
| ICD10 | I80.221 | Phlebitis and thrombophlebitis of right popliteal vein | X | X | — |
| ICD10 | I80.222 | Phlebitis and thrombophlebitis of left popliteal vein | X | X | — |
| ICD10 | I80.223 | Phlebitis and thrombophlebitis of popliteal vein, bilateral | X | X | — |
| ICD10 | I80.229 | Phlebitis and thrombophlebitis of unspecified popliteal vein | X | X | — |
| ICD10 | I80.23 | Phlebitis and thrombophlebitis of right tibial vein | X | X | — |
| ICD10 | I80.231 | Phlebitis and thrombophlebitis of right tibial vein | X | X | — |
| ICD10 | I80.232 | Phlebitis and thrombophlebitis of left tibial vein | X | X | — |
| ICD10 | I80.233 | Phlebitis and thrombophlebitis of tibial vein, bilateral | X | X | — |
| ICD10 | I80.239 | Phlebitis and thrombophlebitis of unspecified tibial vein | X | X | — |
| ICD10 | I80.24 | Phlebitis and thrombophlebitis of right peroneal vein | X | X | — |
| ICD10 | I80.241 | Phlebitis and thrombophlebitis of right peroneal vein | X | X | — |
| ICD10 | I80.242 | Phlebitis and thrombophlebitis of left peroneal vein | X | X | — |
| ICD10 | I80.243 | Phlebitis and thrombophlebitis of peroneal vein, bilateral | X | X | — |
| ICD10 | I80.249 | Phlebitis and thrombophlebitis of unspecified peroneal vein | X | X | — |
| ICD10 | I80.25 | Phlebitis and thrombophlebitis of right calf muscular vein | X | X | — |
| ICD10 | I80.251 | Phlebitis and thrombophlebitis of right calf muscular vein | X | X | — |
| ICD10 | I80.252 | Phlebitis and thrombophlebitis of left calf muscular vein | X | X | — |
| ICD10 | I80.253 | Phlebitis and thrombophlebitis of left calf muscular vein, bilateral | X | X | — |
| ICD10 | I80.259 | Phlebitis and thrombophlebitis of unspecified calf muscular vein | X | X | — |
| ICD10 | I80.29 | Phlebitis and thrombophlebitis of other deep vessels of lower extremities | X | X | — |
| ICD10 | I80.291 | Phlebitis and thrombophlebitis of other deep vessels of right lower extremity | X | X | — |
| ICD10 | I80.292 | Phlebitis and thrombophlebitis of other deep vessels of left lower extremity | X | X | — |
| ICD10 | I80.293 | Phlebitis and thrombophlebitis of other deep vessels of lower extremity, bilateral | X | X | — |
| ICD10 | I80.299 | Phlebitis and thrombophlebitis of other deep vessels of unspecified lower extremity | X | X | — |
| ICD10 | I82.2 | Phlebitis and thrombophlebitis of unspecified peroneal vein | X | X | — |
| ICD10 | I82.21 | Embolism and thrombosis of superior vena cava | X | X | X |
| ICD10 | I82.210 | Acute embolism and thrombosis of superior vena cava | X | X | X |
| ICD10 | I82.22 | Embolism and thrombosis of inferior vena cava | X | X | X |
| ICD10 | I82.220 | Acute embolism and thrombosis of inferior vena cava | X | X | X |
| ICD10 | I82.290 | Acute embolism and thrombosis of other thoracic veins | X | X | X |
| ICD10 | I82.4 | Acute embolism and thrombosis of deep veins of lower extremity | X | X | X |
| ICD10 | I82.40 | Acute embolism and thrombosis of unspecified deep veins of lower extremity | X | X | X |
| ICD10 | I82.401 | Acute embolism and thrombosis of unspecified deep veins of right lower extremity | X | X | X |
| ICD10 | I82.402 | Acute embolism and thrombosis of unspecified deep veins of left lower extremity | X | X | X |
| ICD10 | I82.403 | Acute embolism and thrombosis of unspecified deep veins of lower extremity, bilateral | X | X | X |
| ICD10 | I82.409 | Acute embolism and thrombosis of unspecified deep veins of unspecified lower extremity | X | X | X |
| ICD10 | I82.41 | Acute embolism and thrombosis of femoral vein | X | X | X |
| ICD10 | I82.411 | Acute embolism and thrombosis of right femoral vein | X | X | X |
| ICD10 | I82.412 | Acute embolism and thrombosis of left femoral vein | X | X | X |
| ICD10 | I82.413 | Acute embolism and thrombosis of femoral vein, bilateral | X | X | X |
| ICD10 | I82.419 | Acute embolism and thrombosis of unspecified femoral vein | X | X | X |
| ICD10 | I82.42 | Acute embolism and thrombosis of iliac vein | X | X | X |
| ICD10 | I82.421 | Acute embolism and thrombosis of right iliac vein | X | X | X |
| ICD10 | I82.422 | Acute embolism and thrombosis of left iliac vein | X | X | X |
| ICD10 | I82.423 | Acute embolism and thrombosis of iliac vein, bilateral | X | X | X |
| ICD10 | I82.429 | Acute embolism and thrombosis of unspecified iliac vein | X | X | X |
| ICD10 | I82.43 | Acute embolism and thrombosis of popliteal vein | X | X | X |
| ICD10 | I82.431 | Acute embolism and thrombosis of right popliteal vein | X | X | X |
| ICD10 | I82.432 | Acute embolism and thrombosis of left popliteal vein | X | X | X |
| ICD10 | I82.433 | Acute embolism and thrombosis of popliteal vein, bilateral | X | X | X |
| ICD10 | I82.439 | Acute embolism and thrombosis of unspecified popliteal vein | X | X | X |
| ICD10 | I82.44 | Acute embolism and thrombosis of tibial vein | X | X | X |
| ICD10 | I82.441 | Acute embolism and thrombosis of right tibial vein | X | X | X |
| ICD10 | I82.442 | Acute embolism and thrombosis of left tibial vein | X | X | X |
| ICD10 | I82.443 | Acute embolism and thrombosis of tibial vein, bilateral | X | X | X |
| ICD10 | I82.449 | Acute embolism and thrombosis of unspecified tibial vein | X | X | X |
| ICD10 | I82.45 | Acute embolism and thrombosis of right peroneal vein | X | X | X |
| ICD10 | I82.451 | Acute embolism and thrombosis of right peroneal vein | X | X | X |
| ICD10 | I82.452 | Acute embolism and thrombosis of left peroneal vein | X | X | X |
| ICD10 | I82.453 | Acute embolism and thrombosis of peroneal vein, bilateral | X | X | X |
| ICD10 | I82.459 | Acute embolism and thrombosis of unspecified peroneal vein | X | X | X |
| ICD10 | I82.46 | Acute embolism and thrombosis of right calf muscular vein | X | X | X |
| ICD10 | I82.461 | Acute embolism and thrombosis of right calf muscular vein | X | X | X |
| ICD10 | I82.462 | Acute embolism and thrombosis of left calf muscular vein | X | X | X |
| ICD10 | I82.463 | Acute embolism and thrombosis of calf muscular vein, bilateral | X | X | X |
| ICD10 | I82.469 | Acute embolism and thrombosis of unspecified calf muscular vein | X | X | X |
| ICD10 | I82.49 | Acute embolism and thrombosis of other specified deep vein of lower extremity | X | X | X |
| ICD10 | I82.491 | Acute embolism and thrombosis of other specified deep vein of right lower extremity | X | X | X |
| ICD10 | I82.492 | Acute embolism and thrombosis of other specified deep vein of left lower extremity | X | X | X |
| ICD10 | I82.493 | Acute embolism and thrombosis of other specified deep vein of lower extremity, bilateral | X | X | X |
| ICD10 | I82.499 | Acute embolism and thrombosis of other specified deep vein of unspecified lower extremity | X | X | X |
| ICD10 | I82.4Y | Acute embolism and thrombosis of unspecified deep veins of proximal lower extremity | X | X | X |
| ICD10 | I82.4Y1 | Acute embolism and thrombosis of unspecified deep veins of right proximal lower extremity | X | X | X |
| ICD10 | I82.4Y2 | Acute embolism and thrombosis of unspecified deep veins of left proximal lower extremity | X | X | X |
| ICD10 | I82.4Y3 | Acute embolism and thrombosis of unspecified deep veins of proximal lower extremity, bilateral | X | X | X |
| ICD10 | I82.4Y9 | Acute embolism and thrombosis of unspecified deep veins of unspecified proximal lower extremity | X | X | X |
| ICD10 | I82.4Z | Acute embolism and thrombosis of unspecified deep veins of distal lower extremity | X | X | X |
| ICD10 | I82.4Z1 | Acute embolism and thrombosis of unspecified deep veins of right distal lower extremity | X | X | X |
| ICD10 | I82.4Z2 | Acute embolism and thrombosis of unspecified deep veins of left distal lower extremity | X | X | X |
| ICD10 | I82.4Z3 | Acute embolism and thrombosis of unspecified deep veins of distal lower extremity, bilateral | X | X | X |
| ICD10 | I82.4Z9 | Acute embolism and thrombosis of unspecified deep veins of unspecified distal lower extremity | X | X | X |
| ICD10 | I82.52 | Acute embolism and thrombosis of axillary vein | X | X | X |
| ICD10 | I82.62 | Acute embolism and thrombosis of deep veins of upper extremity | X | X | X |
| ICD10 | I82.621 | Acute embolism and thrombosis of deep veins of right upper extremity | X | X | X |
| ICD10 | I82.622 | Acute embolism and thrombosis of deep veins of left upper extremity | X | X | X |
| ICD10 | I82.623 | Acute embolism and thrombosis of deep veins of upper extremity, bilateral | X | X | X |
| ICD10 | I82.629 | Acute embolism and thrombosis of deep veins of unspecified upper extremity | X | X | X |
| ICD10 | I82.A | Embolism and thrombosis of axillary vein | X | X | X |
| ICD10 | I82.A1 | Acute embolism and thrombosis of axillary vein | X | X | X |
| ICD10 | I82.A11 | Acute embolism and thrombosis of right axillary vein | X | X | X |
| ICD10 | I82.A12 | Acute embolism and thrombosis of left axillary vein | X | X | X |
| ICD10 | I82.A13 | Acute embolism and thrombosis of axillary vein, bilateral | X | X | X |
| ICD10 | I82.A19 | Acute embolism and thrombosis of unspecified axillary vein | X | X | X |
| ICD10 | I82.B | Embolism and thrombosis of subclavian vein | X | X | X |
| ICD10 | I82.B1 | Acute embolism and thrombosis of subclavian vein | X | X | X |
| ICD10 | I82.B11 | Acute embolism and thrombosis of right subclavian vein | X | X | X |
| ICD10 | I82.B12 | Acute embolism and thrombosis of left subclavian vein | X | X | X |
| ICD10 | I82.B13 | Acute embolism and thrombosis of subclavian vein, bilateral | X | X | X |
| ICD10 | I82.B19 | Acute embolism and thrombosis of unspecified subclavian vein | X | X | X |
| ICD10 | I82.C | Embolism and thrombosis of internal jugular vein | X | X | X |
| ICD10 | I82.C1 | Acute embolism and thrombosis of internal jugular vein | X | X | X |
| ICD10 | I82.C11 | Acute embolism and thrombosis of right internal jugular vein | X | X | X |
| ICD10 | I82.C12 | Acute embolism and thrombosis of left internal jugular vein | X | X | X |
| ICD10 | I82.C13 | Acute embolism and thrombosis of internal jugular vein, bilateral | X | X | X |
| ICD10 | I82.C19 | Acute embolism and thrombosis of unspecified internal jugular vein | X | X | X |
| ICD9 | 415.1 | Pulmonary embolism and infarction | X | X | X |
| ICD9 | 415.11 | Iatrogenic pulmonary embolism and infarction | X | X | X |
| ICD9 | 415.13 | Saddle embolus of pulmonary artery | X | X | X |
| ICD9 | 415.19 | Other pulmonary embolism and infarction | X | X | X |
| ICD9 | 451.1 | Phlebitis and thrombophlebitis; of deep vessels of lower extremities | X | X | — |
| ICD9 | 451.11 | Phlebitis and thrombophlebitis of femoral vein (deep) (superficial) | X | X | — |
| ICD9 | 451.19 | Phlebitis and thrombophlebitis of deep veins of lower extremities, other | X | X | — |
| ICD9 | 451.81 | Phlebitis and thrombophlebitis of iliac vein | X | X | — |
| ICD9 | 451.83 | Phlebitis and thrombophlebitis of deep veins of upper extremities | X | X | — |
| ICD9 | 453.2 | Other venous embolism and thrombosis of inferior vena cava | X | X | — |
| ICD9 | 453.4 | Acute venous embolism and thrombosis of deep vessels of lower extremity | X | X | X |
| ICD9 | 453.40 | Acute venous embolism and thrombosis of unspecified deep vessels of lower extremity | X | X | X |
| ICD9 | 453.41 | Acute venous embolism and thrombosis of deep vessels of proximal lower extremity | X | X | X |
| ICD9 | 453.42 | Acute venous embolism and thrombosis of deep vessels of distal lower extremity | X | X | X |
| ICD9 | 453.82 | Acute venous embolism and thrombosis of deep veins of upper extremity | X | X | X |
| ICD9 | 453.84 | Acute venous embolism and thrombosis of axillary veins | X | X | X |
| ICD9 | 453.85 | Acute venous embolism and thrombosis of subclavian veins | X | X | X |
| ICD9 | 453.86 | Acute venous embolism and thrombosis of internal jugular veins | X | X | X |
| ICD9 | 453.87 | Acute venous embolism and thrombosis of other thoracic veins | X | X | X |
| ICD9 | 453.89 | Acute venous embolism and thrombosis of other specified veins | X | X | X |

VTE, venous thromboembolism. “X” represents codes that were included for identification of the endpoint under the specified setting. “—" represents codes that were reviewed with a decision to not include.

## **Identification of the Major Bleeding Endpoint**

Studies investigating anticoagulation therapies, including randomized trials, have utilized a variety of definitions for identifying a major bleeding event. To make the criteria more uniform and facilitate comparison across studies, the International Society on Thrombosis and Haemostasis (ISTH) proposed a standardized definition for major bleeding in 2005. [7] The guiding principle was that the standardized definition must be based on objective criteria, and major bleeds are those that result in death, are life-threatening, cause chronic sequelae, or consume major healthcare resources. The ISTH recommended the following criteria for major bleeding in non-surgical patients:

- Fatal bleeding
- Bleeding in a critical area or organ, such as intracranial, intraspinal, intraocular, retroperitoneal, intraarticular, pericardial, or intramuscular with compartment syndrome
- Bleeding causing a fall in hemoglobin level of 20 g/L (1.24 mmol/L) or more, or leading to transfusion of two or more units of whole blood or red cells

It is largely possible to operationalize the second criterion with information available in the study database, and our adopted algorithm mainly rests on this criterion. Effective operationalization of the first criterion with a US database would require integration with the National Death Index (NDI) database. [8] We have not pursued this option due to considerable added complexity and negligible anticipated impact on results. The number of fatal bleeding events are expected to be low during the 90-day follow-up in the study population, and in addition a vast majority of these fatal bleeding events are expected to meet the definition of the second criterion, thus implicitly being captured in the analysis. Implementation of the third criterion, based on fall in hemoglobin and transfusion, is challenging due to the ambiguities around recording of drop in hemoglobin levels and units of blood transfused in the study database. Majority of the cases meeting the third criterion are also expected to meet the second one. In summary, we do not expect many cases of major bleeding that will only meet the first or third criteria, and not the second one.

Studies evaluating the occurrence of major bleeding in observational studies have mainly considered intracranial and gastrointestinal bleeding. [1-2] Our algorithm offers a substantial improvement on this approach by expanding to critical sites and organs under the ISTH definition (second criterion). Any code indicating bleeding in an area or organ (critical or non-critical) was considered as major bleed if it occurred in the primary position in inpatient setting. A subset of these codes was then considered as major bleeding for inpatient non-primary position. The main requirement for this subset was critical site or organ per second criteria of the ISTH definition. Any code explicitly mentioning “chronic” was not considered for non-primary position, and conversely any code explicitly mentioning “acute” was retained for non-primary position. Codes for compartment syndrome codes were required to have a code for hemorrhage or hematoma during the same hospitalization. Many of the codes in the proposed algorithm, in particular those for intracranial and gastrointestinal bleeding, have been utilized in previous large observational analyses, and reviewed in validation studies, which have indicated a high PPV for these codes for the identification of major bleeding events in inpatient setting, especially for primary position. [1-2,9-10] The justification for being restrictive for codes in inpatient non-primary position is supported by validation studies where codes representing non-critical sites (e.g. gastrointestinal, genitourinary) were demonstrated to have a low positive predictive value for the inpatient non-primary position. [10]

**Table S3. Codes for identification of the major bleeding endpoint.**

| **Code Type** | **Code** | **Description** | **Inpatient Primary** | **Inpatient Non-Primary** |
| --- | --- | --- | --- | --- |
| ICD-10 | H05.23 | Hemorrhage of orbit | X | X |
| ICD-10 | H05.231 | Hemorrhage of right orbit | X | X |
| ICD-10 | H05.232 | Hemorrhage of left orbit | X | X |
| ICD-10 | H05.233 | Hemorrhage of bilateral orbit | X | X |
| ICD-10 | H05.239 | Hemorrhage of unspecified orbit | X | X |
| ICD-10 | H31.3 | Choroidal hemorrhage and rupture | X | X |
| ICD-10 | H31.30 | Unspecified choroidal hemorrhage | X | X |
| ICD-10 | H31.301 | Unspecified choroidal hemorrhage, right eye | X | X |
| ICD-10 | H31.302 | Unspecified choroidal hemorrhage, left eye | X | X |
| ICD-10 | H31.303 | Unspecified choroidal hemorrhage, bilateral | X | X |
| ICD-10 | H31.309 | Unspecified choroidal hemorrhage, unspecified eye | X | X |
| ICD-10 | H31.31 | Expulsive choroidal hemorrhage | X | X |
| ICD-10 | H31.311 | Expulsive choroidal hemorrhage, right eye | X | X |
| ICD-10 | H31.312 | Expulsive choroidal hemorrhage, left eye | X | X |
| ICD-10 | H31.313 | Expulsive choroidal hemorrhage, bilateral | X | X |
| ICD-10 | H31.319 | Expulsive choroidal hemorrhage, unspecified eye | X | X |
| ICD-10 | H35.6 | Retinal hemorrhage | X | X |
| ICD-10 | H35.60 | Retinal hemorrhage, unspecified eye | X | X |
| ICD-10 | H35.61 | Retinal hemorrhage, right eye | X | X |
| ICD-10 | H35.62 | Retinal hemorrhage, left eye | X | X |
| ICD-10 | H35.63 | Retinal hemorrhage, bilateral | X | X |
| ICD-10 | H43.1 | Vitreous hemorrhage | X | X |
| ICD-10 | H43.10 | Vitreous bleeding unspecified eye | X | X |
| ICD-10 | H43.11 | Vitreous bleeding right eye | X | X |
| ICD-10 | H43.12 | Vitreous bleeding left eye | X | X |
| ICD-10 | H43.13 | Vitreous bleeding bilateral | X | X |
| ICD-10 | I31.2 | Hemopericardium, not elsewhere classified | X | X |
| ICD-10 | I60 | Nontraumatic subarachnoid hemorrhage | X | X |
| ICD-10 | I60.0 | Nontraumatic subarachnoid hemorrhage from carotid siphon and bifurcation | X | X |
| ICD-10 | I60.00 | Nontraumatic subarachnoid hemorrhage from unspecified carotid siphon and bifurcation | X | X |
| ICD-10 | I60.01 | Nontraumatic subarachnoid hemorrhage from right carotid siphon and bifurcation | X | X |
| ICD-10 | I60.02 | Nontraumatic subarachnoid hemorrhage from left carotid siphon and bifurcation | X | X |
| ICD-10 | I60.1 | Nontraumatic subarachnoid hemorrhage from middle cerebral artery | X | X |
| ICD-10 | I60.10 | Nontraumatic subarachnoid hemorrhage from unspecified middle cerebral artery | X | X |
| ICD-10 | I60.11 | Nontraumatic subarachnoid hemorrhage from right middle cerebral artery | X | X |
| ICD-10 | I60.12 | Nontraumatic subarachnoid hemorrhage from left middle cerebral artery | X | X |
| ICD-10 | I60.2 | Nontraumatic subarachnoid hemorrhage from anterior communicating artery | X | X |
| ICD-10 | I60.3 | Nontraumatic subarachnoid hemorrhage from posterior communicating artery | X | X |
| ICD-10 | I60.30 | Nontraumatic subarachnoid hemorrhage from unspecified posterior communicating artery | X | X |
| ICD-10 | I60.31 | Nontraumatic subarachnoid hemorrhage from right posterior communicating artery | X | X |
| ICD-10 | I60.32 | Nontraumatic subarachnoid hemorrhage from left posterior communicating artery | X | X |
| ICD-10 | I60.4 | Nontraumatic subarachnoid hemorrhage from basilar artery | X | X |
| ICD-10 | I60.5 | Nontraumatic subarachnoid hemorrhage from vertebral artery | X | X |
| ICD-10 | I60.50 | Nontraumatic subarachnoid hemorrhage from unspecified vertebral artery | X | X |
| ICD-10 | I60.51 | Nontraumatic subarachnoid hemorrhage from right vertebral artery | X | X |
| ICD-10 | I60.52 | Nontraumatic subarachnoid hemorrhage from left vertebral artery | X | X |
| ICD-10 | I60.6 | Nontraumatic subarachnoid hemorrhage from other intracranial arteries | X | X |
| ICD-10 | I60.7 | Nontraumatic subarachnoid hemorrhage from unspecified intracranial artery | X | X |
| ICD-10 | I60.8 | Other nontraumatic subarachnoid hemorrhage | X | X |
| ICD-10 | I60.9 | Nontraumatic subarachnoid hemorrhage, unspecified | X | X |
| ICD-10 | I61 | Nontraumatic intracerebral hemorrhage | X | X |
| ICD-10 | I61.0 | Nontraumatic intracerebral hemorrhage in hemisphere, subcortical | X | X |
| ICD-10 | I61.1 | Nontraumatic intracerebral hemorrhage in hemisphere, cortical | X | X |
| ICD-10 | I61.2 | Nontraumatic intracerebral hemorrhage in hemisphere, unspecified | X | X |
| ICD-10 | I61.3 | Nontraumatic intracerebral hemorrhage in brain stem | X | X |
| ICD-10 | I61.4 | Nontraumatic intracerebral hemorrhage in cerebellum | X | X |
| ICD-10 | I61.5 | Nontraumatic intracerebral hemorrhage, intraventricular | X | X |
| ICD-10 | I61.6 | Nontraumatic intracerebral hemorrhage, multiple localized | X | X |
| ICD-10 | I61.8 | Other nontraumatic intracerebral hemorrhage | X | X |
| ICD-10 | I61.9 | Nontraumatic intracerebral hemorrhage, unspecified | X | X |
| ICD-10 | I62 | Other and unspecified nontraumatic intracranial hemorrhage | X | X |
| ICD-10 | I62.0 | Nontraumatic subdural hemorrhage | X | X |
| ICD-10 | I62.00 | Nontraumatic subdural hemorrhage unspecified | X | X |
| ICD-10 | I62.01 | Nontraumatic acute subdural hemorrhage | X | X |
| ICD-10 | I62.02 | Nontraumatic subacute subdural hemorrhage | X | X |
| ICD-10 | I62.1 | Nontraumatic extradural hemorrhage | X | X |
| ICD-10 | I62.9 | Nontraumatic intracranial hemorrhage, unspecified | X | X |
| ICD-10 | I71.3 | Abdominal aortic aneurysm, ruptured | X | X |
| ICD-10 | I71.30 | Abdominal aortic aneurysm, ruptured, unspecified | X | X |
| ICD-10 | I71.31 | Pararenal abdominal aortic aneurysm, ruptured | X | X |
| ICD-10 | I71.32 | Juxtarenal abdominal aortic aneurysm, ruptured | X | X |
| ICD-10 | I71.33 | Infrarenal abdominal aortic aneurysm, ruptured | X | X |
| ICD-10 | I71.5 | Thoracoabdominal aortic aneurysm, ruptured | X | X |
| ICD-10 | I71.50 | Thoracoabdominal aortic aneurysm, ruptured, unspecified | X | X |
| ICD-10 | I71.51 | Supraceliac aneurysm of the abdominal aorta, ruptured | X | X |
| ICD-10 | I71.52 | Paravisceral aneurysm of the abdominal aorta, ruptured | X | X |
| ICD-10 | I71.8 | Aortic aneurysm of unspecified site, ruptured | X | X |
| ICD-10 | I85.01 | Esophageal varices with bleeding | X | — |
| ICD-10 | I85.11 | Secondary esophageal varices with bleeding | X | — |
| ICD-10 | K20.81 | Other esophagitis with bleeding | X | — |
| ICD-10 | K20.91 | Esophagitis, unspecified with bleeding | X | — |
| ICD-10 | K22.11 | Ulcer of oesophagus, with bleeding | X | — |
| ICD-10 | K22.6 | Gastro-esophageal laceration-hemorrhage syndrome | X | — |
| ICD-10 | K25.0 | Gastric ulcer, acute with bleeding | X | X |
| ICD-10 | K25.2 | Gastric ulcer, acute with both bleeding and perforation | X | X |
| ICD-10 | K25.4 | Gastric ulcer, chronic or unspecified with bleeding | X | — |
| ICD-10 | K25.6 | Gastric ulcer, acute with both bleeding and perforation | X | X |
| ICD-10 | K26.0 | Duodenal ulcer, acute with bleeding | X | X |
| ICD-10 | K26.2 | Acute duodenal ulcer with both hemorrhage and perforation | X | X |
| ICD-10 | K26.4 | Chronic or unspecified duodenal ulcer with hemorrhage | X | — |
| ICD-10 | K26.6 | Chronic or unspecified duodenal ulcer with both hemorrhage and perforation | X | — |
| ICD-10 | K27.0 | Peptic ulcer, acute with bleeding | X | X |
| ICD-10 | K27.2 | Acute peptic ulcer, site unspecified, with both hemorrhage and perforation | X | X |
| ICD-10 | K27.4 | Chronic or unspecified peptic ulcer, site unspecified, with hemorrhage | X | — |
| ICD-10 | K27.6 | Chronic or unspecified peptic ulcer, site unspecified, with both hemorrhage and perforation | X | — |
| ICD-10 | K28.0 | Gastrojejunal ulcer, acute with bleeding | X | X |
| ICD-10 | K28.2 | Gastrojejunal ulcer, acute with both bleeding and perforation | X | X |
| ICD-10 | K28.4 | Gastrojejunal ulcer, chronic or unspecified with bleeding | X | — |
| ICD-10 | K28.6 | Gastrojejunal ulcer, chronic or unspecified with both bleeding and perforation | X | — |
| ICD-10 | K29.01 | Acute gastritis with bleeding | X | X |
| ICD-10 | K29.21 | Alcoholic gastritis with bleeding | X | — |
| ICD-10 | K29.31 | Chronic superficial gastritis with bleeding | X | — |
| ICD-10 | K29.41 | Chronic atrophic gastritis with bleeding | X | — |
| ICD-10 | K29.51 | Unspecified chronic gastritis with bleeding | X | — |
| ICD-10 | K29.61 | Other gastritis with bleeding | X | — |
| ICD-10 | K29.71 | Gastritis, unspecified with bleeding | X | — |
| ICD-10 | K29.81 | Duodenitis with bleeding | X | — |
| ICD-10 | K29.91 | Gastroduodenitis, unspecified with bleeding | X | — |
| ICD-10 | K31.811 | Angiodysplasia of stomach and duodenum with bleeding | X | — |
| ICD-10 | K31.82 | Dieulafoy lesion (hemorrhagic) of stomach and duodenum | X | — |
| ICD-10 | K55.21 | Angiodysplasia of colon with hemorrhage | X | — |
| ICD-10 | K57.01 | Diverticulitis of small intestine with perforation and abscess with bleeding | X | — |
| ICD-10 | K57.11 | Diverticulosis of small intestine without perforation or abscess with bleeding | X | — |
| ICD-10 | K57.13 | Diverticulitis of small intestine without perforation or abscess with bleeding | X | — |
| ICD-10 | K57.21 | Diverticulitis of large intestine with perforation and abscess with bleeding | X | — |
| ICD-10 | K57.31 | Diverticulosis of large intestine without perforation or abscess with bleeding | X | — |
| ICD-10 | K57.33 | Diverticulitis of large intestine without perforation or abscess with bleeding | X | — |
| ICD-10 | K57.41 | Diverticulitis of both small and large intestine with perforation and abscess with bleeding | X | — |
| ICD-10 | K57.51 | Diverticulosis of both small and large intestine without perforation or abscess with bleeding | X | — |
| ICD-10 | K57.53 | Diverticulitis of both small and large intestine without perforation or abscess with bleeding | X | — |
| ICD-10 | K57.81 | Diverticulitis of intestine, part unspecified, with perforation and abscess with bleeding | X | — |
| ICD-10 | K57.91 | Diverticulosis of intestine, part unspecified, without perforation or abscess with bleeding | X | — |
| ICD-10 | K57.93 | Diverticulitis of intestine, part unspecified, without perforation or abscess with bleeding | X | — |
| ICD-10 | K62.5 | Hemorrhage of anus and rectum | X | — |
| ICD-10 | K66.1 | Haemoperitoneum | X | X |
| ICD-10 | K92.0 | Hematemesis | X | — |
| ICD-10 | K92.1 | Melena | X | — |
| ICD-10 | K92.2 | Gastrointestinal bleeding, unspecified | X | — |
| ICD-10 | M25.0 | Hemarthrosis | X | — |
| ICD-10 | M25.00 | Hemarthrosis, unspecified joint | X | — |
| ICD-10 | M25.01 | Hemarthrosis, shoulder | X | — |
| ICD-10 | M25.011 | Hemarthrosis, right shoulder | X | — |
| ICD-10 | M25.012 | Hemarthrosis, left shoulder | X | — |
| ICD-10 | M25.019 | Hemarthrosis, unspecified shoulder | X | — |
| ICD-10 | M25.02 | Hemarthrosis, elbow | X | — |
| ICD-10 | M25.021 | Hemarthrosis, right elbow | X | — |
| ICD-10 | M25.022 | Hemarthrosis, left elbow | X | — |
| ICD-10 | M25.029 | Hemarthrosis, unspecified elbow | X | — |
| ICD-10 | M25.03 | Hemarthrosis, wrist | X | — |
| ICD-10 | M25.031 | Hemarthrosis, right wrist | X | — |
| ICD-10 | M25.032 | Hemarthrosis, left wrist | X | — |
| ICD-10 | M25.039 | Hemarthrosis, unspecified wrist | X | — |
| ICD-10 | M25.04 | Hemarthrosis, hand | X | — |
| ICD-10 | M25.041 | Hemarthrosis, right hand | X | — |
| ICD-10 | M25.042 | Hemarthrosis, left hand | X | — |
| ICD-10 | M25.049 | Hemarthrosis, unspecified hand | X | — |
| ICD-10 | M25.05 | Hemarthrosis, hip | X | — |
| ICD-10 | M25.051 | Hemarthrosis, right hip | X | — |
| ICD-10 | M25.052 | Hemarthrosis, left hip | X | — |
| ICD-10 | M25.059 | Hemarthrosis, unspecified hip | X | — |
| ICD-10 | M25.06 | Hemarthrosis, knee | X | — |
| ICD-10 | M25.061 | Hemarthrosis, right knee | X | — |
| ICD-10 | M25.062 | Hemarthrosis, left knee | X | — |
| ICD-10 | M25.069 | Hemarthrosis, unspecified knee | X | — |
| ICD-10 | M25.07 | Hemarthrosis, ankle and foot | X | — |
| ICD-10 | M25.071 | Hemarthrosis, right ankle | X | — |
| ICD-10 | M25.072 | Hemarthrosis, left ankle | X | — |
| ICD-10 | M25.073 | Hemarthrosis, unspecified ankle | X | — |
| ICD-10 | M25.074 | Hemarthrosis, right foot | X | — |
| ICD-10 | M25.075 | Hemarthrosis, left foot | X | — |
| ICD-10 | M25.076 | Hemarthrosis, unspecified foot | X | — |
| ICD-10 | M25.08 | Hemarthrosis, other specified site | X | — |
| ICD-10 | M79.81 | Nontraumatic hematoma of soft tissue | X | — |
| ICD-10 | M79.A | Nontraumatic compartment syndrome | X | X |
| ICD-10 | M79.A1 | Nontraumatic compartment syndrome of upper extremity | X | X |
| ICD-10 | M79.A11 | Nontraumatic compartment syndrome of right upper extremity | X | X |
| ICD-10 | M79.A12 | Nontraumatic compartment syndrome of left upper extremity | X | X |
| ICD-10 | M79.A19 | Nontraumatic compartment syndrome of unspecified upper extremity | X | X |
| ICD-10 | M79.A2 | Nontraumatic compartment syndrome of lower extremity | X | X |
| ICD-10 | M79.A21 | Nontraumatic compartment syndrome of right lower extremity | X | X |
| ICD-10 | M79.A22 | Nontraumatic compartment syndrome of left lower extremity | X | X |
| ICD-10 | M79.A29 | Nontraumatic compartment syndrome of unspecified lower extremity | X | X |
| ICD-10 | N02 | Recurrent and persistent hematuria | X | — |
| ICD-10 | N02.0 | Recurrent and persistent hematuria with minor glomerular abnormality | X | — |
| ICD-10 | N02.2 | Recurrent and persistent hematuria with diffuse membranous glomerulonephritis | X | — |
| ICD-10 | N02.3 | Recurrent and persistent hematuria with diffuse mesangial proliferative glomerulonephritis | X | — |
| ICD-10 | N02.4 | Recurrent and persistent hematuria with diffuse endocapillary proliferative glomerulonephritis | X | — |
| ICD-10 | N02.5 | Recurrent and persistent hematuria with diffuse mesangiocapillary glomerulonephritis | X | — |
| ICD-10 | N02.7 | Recurrent and persistent hematuria with diffuse crescentic glomerulonephritis | X | — |
| ICD-10 | N02.8 | Recurrent and persistent hematuria with other morphologic changes | X | — |
| ICD-10 | N02.9 | Recurrent and persistent hematuria with unspecified morphologic changes | X | — |
| ICD-10 | N02.A | Recurrent and persistent hematuria with C3 glomerulonephritis | X | — |
| ICD-10 | N30.01 | Acute cystitis with hematuria | X | — |
| ICD-10 | N30.11 | Interstitial cystitis (chronic) with hematuria | X | — |
| ICD-10 | N30.21 | Other chronic cystitis with hematuria | X | — |
| ICD-10 | N30.31 | Trigonitis with hematuria | X | — |
| ICD-10 | N30.41 | Irradiation cystitis with hematuria | X | — |
| ICD-10 | N30.81 | Other cystitis with hematuria | X | — |
| ICD-10 | N30.91 | Cystitis, unspecified with hematuria | X | — |
| ICD-10 | N42.1 | Congestion and hemorrhage of prostate | X | — |
| ICD-10 | N83.7 | Hematoma of broad ligament | X | — |
| ICD-10 | N836 | Haematosalpinx | X | — |
| ICD-10 | N857 | Haematometra | X | — |
| ICD-10 | N897 | Haematocolpos | X | — |
| ICD-10 | N92.4 | Excessive bleeding in the premenopausal period | X | — |
| ICD-10 | N93.8 | Other specified abnormal uterine and vaginal bleeding | X | — |
| ICD-10 | N93.9 | Abnormal uterine and vaginal bleeding, unspecified | X | — |
| ICD-10 | N95.0 | Postmenopausal bleeding | X | — |
| ICD-10 | O051 | Other abortion; Incomplete, complicated by delayed or excessive haemorrhage | X | — |
| ICD-10 | O208 | Other haemorrhage in early pregnancy | X | — |
| ICD-10 | O209 | Haemorrhage in early pregnancy, unspecified | X | — |
| ICD-10 | O224 | Haemorrhoids in pregnancy | X | — |
| ICD-10 | O460 | Antepartum haemorrhage with coagulation defect | X | — |
| ICD-10 | O468 | Other antepartum haemorrhage | X | — |
| ICD-10 | O469 | Antepartum haemorrhage, unspecified | X | — |
| ICD-10 | O670 | Intrapartum haemorrhage with coagulation defect | X | — |
| ICD-10 | O678 | Other intrapartum haemorrhage | X | — |
| ICD-10 | O679 | Intrapartum haemorrhage, unspecified | X | — |
| ICD-10 | O720 | Third-stage haemorrhage | X | — |
| ICD-10 | O721 | Other immediate postpartum haemorrhage | X | — |
| ICD-10 | O722 | Delayed and secondary postpartum haemorrhage | X | — |
| ICD-10 | R04.0 | Epistaxis | X | — |
| ICD-10 | R04.1 | Hemorrhage from throat | X | — |
| ICD-10 | R04.2 | Haemoptysis | X | — |
| ICD-10 | R04.89 | Hemorrhage from other sites in respiratory passages | X | — |
| ICD-10 | R04.9 | Hemorrhage from respiratory passages, unspecified | X | — |
| ICD-10 | R31.0 | Gross hematuria | X | — |
| ICD-10 | S06.340A | Traumatic hemorrhage of right cerebrum without loss of consciousness, initial encounter | X | X |
| ICD-10 | S06.341A | Traumatic hemorrhage of right cerebrum with loss of consciousness of 30 minutes or less, initial encounter | X | X |
| ICD-10 | S06.342A | Traumatic hemorrhage of right cerebrum with loss of consciousness of 31 minutes to 59 minutes, initial encounter | X | X |
| ICD-10 | S06.343A | Traumatic hemorrhage of right cerebrum with loss of consciousness of 1 hours to 5 hours 59 minutes, initial encounter | X | X |
| ICD-10 | S06.344A | Traumatic hemorrhage of right cerebrum with loss of consciousness of 6 hours to 24 hours, initial encounter | X | X |
| ICD-10 | S06.345A | Traumatic hemorrhage of right cerebrum with loss of consciousness greater than 24 hours with return to pre-existing conscious level, initial encounter | X | X |
| ICD-10 | S06.346A | Traumatic hemorrhage of right cerebrum with loss of consciousness greater than 24 hours without return to pre-existing conscious level with patient surviving, initial encounter | X | X |
| ICD-10 | S06.347A | Traumatic hemorrhage of right cerebrum with loss of consciousness of any duration with death due to brain injury prior to regaining consciousness, initial encounter | X | X |
| ICD-10 | S06.348A | Traumatic hemorrhage of right cerebrum with loss of consciousness of any duration with death due to other cause prior to regaining consciousness, initial encounter | X | X |
| ICD-10 | S06.349A | Traumatic hemorrhage of right cerebrum with loss of consciousness of unspecified duration, initial encounter | X | X |
| ICD-10 | S06.34AA | Traumatic hemorrhage of right cerebrum with loss of consciousness status unknown, initial encounter | X | X |
| ICD-10 | S06.350A | Traumatic hemorrhage of left cerebrum without loss of consciousness, initial encounter | X | X |
| ICD-10 | S06.351A | Traumatic hemorrhage of left cerebrum with loss of consciousness of 30 minutes or less, initial encounter | X | X |
| ICD-10 | S06.352A | Traumatic hemorrhage of left cerebrum with loss of consciousness of 31 minutes to 59 minutes, initial encounter | X | X |
| ICD-10 | S06.353A | Traumatic hemorrhage of left cerebrum with loss of consciousness of 1 hours to 5 hours 59 minutes, initial encounter | X | X |
| ICD-10 | S06.354A | Traumatic hemorrhage of left cerebrum with loss of consciousness of 6 hours to 24 hours, initial encounter | X | X |
| ICD-10 | S06.355A | Traumatic hemorrhage of left cerebrum with loss of consciousness greater than 24 hours with return to pre-existing conscious level, initial encounter | X | X |
| ICD-10 | S06.356A | Traumatic hemorrhage of left cerebrum with loss of consciousness greater than 24 hours without return to pre-existing conscious level with patient surviving, initial encounter | X | X |
| ICD-10 | S06.357A | Traumatic hemorrhage of left cerebrum with loss of consciousness of any duration with death due to brain injury prior to regaining consciousness, initial encounter | X | X |
| ICD-10 | S06.358A | Traumatic hemorrhage of left cerebrum with loss of consciousness of any duration with death due to other cause prior to regaining consciousness, initial encounter | X | X |
| ICD-10 | S06.359A | Traumatic hemorrhage of left cerebrum with loss of consciousness of unspecified duration, initial encounter | X | X |
| ICD-10 | S06.35AA | Traumatic hemorrhage of left cerebrum with loss of consciousness status unknown, initial encounter | X | X |
| ICD-10 | S06.360A | Traumatic hemorrhage of cerebrum, unspecified, without loss of consciousness, initial encounter | X | X |
| ICD-10 | S06.361A | Traumatic hemorrhage of cerebrum, unspecified, with loss of consciousness of 30 minutes or less, initial encounter | X | X |
| ICD-10 | S06.362A | Traumatic hemorrhage of cerebrum, unspecified, with loss of consciousness of 31 minutes to 59 minutes, initial encounter | X | X |
| ICD-10 | S06.363A | Traumatic hemorrhage of cerebrum, unspecified, with loss of consciousness of 1 hours to 5 hours 59 minutes, initial encounter | X | X |
| ICD-10 | S06.364A | Traumatic hemorrhage of cerebrum, unspecified, with loss of consciousness of 6 hours to 24 hours, initial encounter | X | X |
| ICD-10 | S06.365A | Traumatic hemorrhage of cerebrum, unspecified, with loss of consciousness greater than 24 hours with return to pre-existing conscious level, initial encounter | X | X |
| ICD-10 | S06.366A | Traumatic hemorrhage of cerebrum, unspecified, with loss of consciousness greater than 24 hours without return to pre-existing conscious level with patient surviving, initial encounter | X | X |
| ICD-10 | S06.367A | Traumatic hemorrhage of cerebrum, unspecified, with loss of consciousness of any duration with death due to brain injury prior to regaining consciousness, initial encounter | X | X |
| ICD-10 | S06.368A | Traumatic hemorrhage of cerebrum, unspecified, with loss of consciousness of any duration with death due to other cause prior to regaining consciousness, initial encounter | X | X |
| ICD-10 | S06.369A | Traumatic hemorrhage of cerebrum, unspecified, with loss of consciousness of unspecified duration, initial encounter | X | X |
| ICD-10 | S06.36AA | Traumatic hemorrhage of cerebrum, unspecified, with loss of consciousness status unknown, initial encounter | X | X |
| ICD-10 | S06.4X0A | Epidural hemorrhage without loss of consciousness, initial encounter | X | X |
| ICD-10 | S06.4X1A | Epidural hemorrhage with loss of consciousness of 30 minutes or less, initial encounter | X | X |
| ICD-10 | S06.4X2A | Epidural hemorrhage with loss of consciousness of 31 minutes to 59 minutes, initial encounter | X | X |
| ICD-10 | S06.4X3A | Epidural hemorrhage with loss of consciousness of 1 hour to 5 hours 59 minutes, initial encounter | X | X |
| ICD-10 | S06.4X4A | Epidural hemorrhage with loss of consciousness of 6 hours to 24 hours, initial encounter | X | X |
| ICD-10 | S06.4X5A | Epidural hemorrhage with loss of consciousness greater than 24 hours with return to pre-existing conscious level, initial encounter | X | X |
| ICD-10 | S06.4X6A | Epidural hemorrhage with loss of consciousness greater than 24 hours without return to pre-existing conscious level with patient surviving, initial encounter | X | X |
| ICD-10 | S06.4X7A | Epidural hemorrhage with loss of consciousness of any duration with death due to brain injury prior to regaining consciousness, initial encounter | X | X |
| ICD-10 | S06.4X8A | Epidural hemorrhage with loss of consciousness of any duration with death due to other causes prior to regaining consciousness, initial encounter | X | X |
| ICD-10 | S06.4X9A | Epidural hemorrhage with loss of consciousness of unspecified duration, initial encounter | X | X |
| ICD-10 | S06.4XAA | Epidural hemorrhage with loss of consciousness status unknown, initial encounter | X | X |
| ICD-10 | S06.5X0A | Traumatic subdural hemorrhage without loss of consciousness, initial encounter | X | X |
| ICD-10 | S06.5X1A | Traumatic subdural hemorrhage with loss of consciousness of 30 minutes or less, initial encounter | X | X |
| ICD-10 | S06.5X2A | Traumatic subdural hemorrhage with loss of consciousness of 31 minutes to 59 minutes, initial encounter | X | X |
| ICD-10 | S06.5X3A | Traumatic subdural hemorrhage with loss of consciousness of 1 hour to 5 hours 59 minutes, initial encounter | X | X |
| ICD-10 | S06.5X4A | Traumatic subdural hemorrhage with loss of consciousness of 6 hours to 24 hours, initial encounter | X | X |
| ICD-10 | S06.5X5A | Traumatic subdural hemorrhage with loss of consciousness greater than 24 hours with return to pre-existing conscious level, initial encounter | X | X |
| ICD-10 | S06.5X6A | Traumatic subdural hemorrhage with loss of consciousness greater than 24 hours without return to pre-existing conscious level with patient surviving, initial encounter | X | X |
| ICD-10 | S06.5X7A | Traumatic subdural hemorrhage with loss of consciousness of any duration with death due to brain injury before regaining consciousness, initial encounter | X | X |
| ICD-10 | S06.5X8A | Traumatic subdural hemorrhage with loss of consciousness of any duration with death due to other cause before regaining consciousness, initial encounter | X | X |
| ICD-10 | S06.5X9A | Traumatic subdural hemorrhage with loss of consciousness of unspecified duration, initial encounter | X | X |
| ICD-10 | S06.5XAA | Traumatic subdural hemorrhage with loss of consciousness of any duration with death due to other cause before regaining consciousness, initial encounter | X | X |
| ICD-10 | S06.6X0A | Traumatic subarachnoid hemorrhage without loss of consciousness, initial encounter | X | X |
| ICD-10 | S06.6X1A | Traumatic subarachnoid hemorrhage with loss of consciousness of 30 minutes or less, initial encounter | X | X |
| ICD-10 | S06.6X2A | Traumatic subarachnoid hemorrhage with loss of consciousness of 31 minutes to 59 minutes, initial encounte | X | X |
| ICD-10 | S06.6X3A | Traumatic subarachnoid hemorrhage with loss of consciousness of 1 hour to 5 hours 59 minutes, initial encounter | X | X |
| ICD-10 | S06.6X4A | Traumatic subarachnoid hemorrhage with loss of consciousness of 6 hours to 24 hours, initial encounter | X | X |
| ICD-10 | S06.6X5A | Traumatic subarachnoid hemorrhage with loss of consciousness greater than 24 hours with return to pre-existing conscious level, initial encounter | X | X |
| ICD-10 | S06.6X6A | Traumatic subarachnoid hemorrhage with loss of consciousness greater than 24 hours without return to pre-existing conscious level with patient surviving, initial encounter | X | X |
| ICD-10 | S06.6X7A | Traumatic subarachnoid hemorrhage with loss of consciousness of any duration with death due to brain injury prior to regaining consciousness, initial encounter | X | X |
| ICD-10 | S06.6X8A | Traumatic subarachnoid hemorrhage with loss of consciousness of any duration with death due to other cause prior to regaining consciousness, initial encounter | X | X |
| ICD-10 | S06.6X9A | Traumatic subarachnoid hemorrhage with loss of consciousness of unspecified duration, initial encounter | X | X |
| ICD-10 | S06.6XAA | Traumatic subarachnoid hemorrhage with loss of consciousness status unknown, initial encounter | X | X |
| ICD-10 | S271 | Traumatic haemothorax | X | X |
| ICD-10 | T79.2XXA | Traumatic secondary and recurrent hemorrhage and seroma, initial encounter | X | X |
| ICD-10 | T79.A | Traumatic compartment syndrome | X | X |
| ICD-10 | T79.A1 | Traumatic compartment syndrome of upper extremity | X | X |
| ICD-10 | T79.A11 | Traumatic compartment syndrome of right upper extremity | X | X |
| ICD-10 | T79.A11A | Traumatic compartment syndrome of right upper extremity, initial encounter | X | X |
| ICD-10 | T79.A12 | Traumatic compartment syndrome of left upper extremity | X | X |
| ICD-10 | T79.A12A | Traumatic compartment syndrome of left upper extremity, initial encounter | X | X |
| ICD-10 | T79.A19 | Traumatic compartment syndrome of unspecified upper extremity | X | X |
| ICD-10 | T79.A19A | Traumatic compartment syndrome of unspecified upper extremity, initial encounter | X | X |
| ICD-10 | T79.A2 | Traumatic compartment syndrome of lower extremity | X | X |
| ICD-10 | T79.A21 | Traumatic compartment syndrome of right lower extremity | X | X |
| ICD-10 | T79.A21A | Traumatic compartment syndrome of right lower extremity, initial encounter | X | X |
| ICD-10 | T79.A22 | Traumatic compartment syndrome of left lower extremity | X | X |
| ICD-10 | T79.A22A | Traumatic compartment syndrome of left lower extremity, initial encounter | X | X |
| ICD-10 | T79.A29 | Traumatic compartment syndrome of unspecified lower extremity | X | X |
| ICD-10 | T79.A29A | Traumatic compartment syndrome of unspecified lower extremity, initial encounter | X | X |
| ICD-10 | T79.A3 | Traumatic compartment syndrome of abdomen | X | X |
| ICD-10 | T79.A3XA | Traumatic compartment syndrome of abdomen, initial encounter | X | X |
| ICD-10 | T79.A9 | Traumatic compartment syndrome of other sites | X | X |
| ICD-10 | T79.A9XA | Traumatic compartment syndrome of other sites, initial encounter | X | X |
| ICD-10 | T810 | Haemorrhage and haematoma complicating a procedure, not elsewhere classified | X | — |
| ICD-9 | 362.81 | Retinal hemorrhage | X | X |
| ICD-9 | 363.6 | Choroidal hemorrhage and rupture | X | X |
| ICD-9 | 363.61 | Choroidal hemorrhage, unspecified | X | X |
| ICD-9 | 363.62 | Expulsive choroidal hemorrhage | X | X |
| ICD-9 | 376.32 | Orbital hemorrhage | X | X |
| ICD-9 | 377.42 | Hemorrhage in optic nerve sheaths | X | X |
| ICD-9 | 379.23 | Vitreous hemorrhage | X | X |
| ICD-9 | 423.0 | Hemopericardium | X | X |
| ICD-9 | 430 | Subarachnoid hemorrhage | X | X |
| ICD-9 | 431 | Intracerebral hemorrhage | X | X |
| ICD-9 | 432 | Other and unspecified intracranial hemorrhage | X | X |
| ICD-9 | 432.0 | Nontraumatic extradural hemorrhage | X | X |
| ICD-9 | 432.1 | Subdural hemorrhage | X | X |
| ICD-9 | 432.9 | Unspecified intracranial hemorrhage | X | X |
| ICD-9 | 456.0 | Esophageal varices with bleeding | X | — |
| ICD-9 | 456.20 | Esophageal varices in diseases classified elsewhere, with bleeding | X | — |
| ICD-9 | 530.21 | Ulcer of esophagus with bleeding | X | — |
| ICD-9 | 530.7 | Gastroesophageal laceration-hemorrhage syndrome | X | — |
| ICD-9 | 530.82 | Esophageal hemorrhage | X | — |
| ICD-9 | 531.0 | Acute gastric ulcer with hemorrhage | X | X |
| ICD-9 | 531.00 | Acute gastric ulcer with hemorrhage, without mention of obstruction | X | X |
| ICD-9 | 531.01 | Acute gastric ulcer with hemorrhage, with obstruction | X | X |
| ICD-9 | 531.2 | Acute gastric ulcer with hemorrhage and perforation | X | X |
| ICD-9 | 531.20 | Acute gastric ulcer with hemorrhage and perforation, without mention of obstruction | X | X |
| ICD-9 | 531.21 | Acute gastric ulcer with hemorrhage and perforation, with obstruction | X | X |
| ICD-9 | 531.4 | Chronic or unspecified gastric ulcer with hemorrhage | X | — |
| ICD-9 | 531.40 | Chronic or unspecified gastric ulcer with hemorrhage, without mention of obstruction | X | — |
| ICD-9 | 531.41 | Chronic or unspecified gastric ulcer with hemorrhage, with obstruction | X | — |
| ICD-9 | 531.6 | Chronic or unspecified gastric ulcer with hemorrhage and perforation | X | — |
| ICD-9 | 531.60 | Chronic or unspecified gastric ulcer with hemorrhage and perforation, without mention of obstruction | X | — |
| ICD-9 | 531.61 | Chronic or unspecified gastric ulcer with hemorrhage and perforation, with obstruction | X | — |
| ICD-9 | 532.0 | Acute duodenal ulcer with hemorrhage | X | X |
| ICD-9 | 532.00 | Acute duodenal ulcer with hemorrhage, without mention of obstruction | X | X |
| ICD-9 | 532.01 | Acute duodenal ulcer with hemorrhage, with obstruction | X | X |
| ICD-9 | 532.2 | Acute duodenal ulcer with hemorrhage and perforation | X | X |
| ICD-9 | 532.20 | Acute duodenal ulcer with hemorrhage and perforation, without mention of obstruction | X | X |
| ICD-9 | 532.21 | Acute duodenal ulcer with hemorrhage and perforation, with obstruction | X | X |
| ICD-9 | 532.4 | Chronic or unspecified duodenal ulcer with hemorrhage | X | — |
| ICD-9 | 532.40 | Chronic or unspecified duodenal ulcer with hemorrhage, without mention of obstruction | X | — |
| ICD-9 | 532.41 | Chronic or unspecified duodenal ulcer with hemorrhage, with obstruction | X | — |
| ICD-9 | 532.6 | Chronic or unspecified duodenal ulcer with hemorrhage and perforation | X | — |
| ICD-9 | 532.60 | Chronic or unspecified duodenal ulcer with hemorrhage and perforation, without mention of obstruction | X | — |
| ICD-9 | 532.61 | Chronic or unspecified duodenal ulcer with hemorrhage and perforation, with obstruction | X | — |
| ICD-9 | 533.0 | Acute peptic ulcer of unspecified site with hemorrhage | X | X |
| ICD-9 | 533.00 | Acute peptic ulcer of unspecified site with hemorrhage, without mention of obstruction | X | X |
| ICD-9 | 533.01 | Acute peptic ulcer of unspecified site with hemorrhage, with obstruction | X | X |
| ICD-9 | 533.2 | Acute peptic ulcer of unspecified site with hemorrhage and perforation | X | X |
| ICD-9 | 533.20 | Acute peptic ulcer of unspecified site with hemorrhage and perforation, without mention of obstruction | X | X |
| ICD-9 | 533.21 | Acute peptic ulcer of unspecified site with hemorrhage and perforation, with obstruction | X | X |
| ICD-9 | 533.4 | Chronic or unspecified peptic ulcer of unspecified site with hemorrhage | X | — |
| ICD-9 | 533.40 | Chronic or unspecified peptic ulcer of unspecified site with hemorrhage, without mention of obstruction | X | — |
| ICD-9 | 533.41 | Chronic or unspecified peptic ulcer of unspecified site with hemorrhage, with obstruction | X | — |
| ICD-9 | 533.6 | Chronic or unspecified peptic ulcer of unspecified site with hemorrhage and perforation | X | — |
| ICD-9 | 533.60 | Chronic or unspecified peptic ulcer of unspecified site with hemorrhage and perforation, without mention of obstruction | X | — |
| ICD-9 | 533.61 | Chronic or unspecified peptic ulcer of unspecified site with hemorrhage and perforation, with obstruction | X | — |
| ICD-9 | 534.0 | Acute gastrojejunal ulcer with hemorrhage | X | X |
| ICD-9 | 534.00 | Acute gastrojejunal ulcer with hemorrhage, without mention of obstruction | X | X |
| ICD-9 | 534.01 | Acute gastrojejunal ulcer, with hemorrhage, with obstruction | X | X |
| ICD-9 | 534.2 | Acute gastrojejunal ulcer with hemorrhage and perforation | X | X |
| ICD-9 | 534.20 | Acute gastrojejunal ulcer with hemorrhage and perforation, without mention of obstruction | X | X |
| ICD-9 | 534.21 | Acute gastrojejunal ulcer with hemorrhage and perforation, with obstruction | X | X |
| ICD-9 | 534.4 | Chronic or unspecified gastrojejunal ulcer with hemorrhage | X | — |
| ICD-9 | 534.40 | Chronic or unspecified gastrojejunal ulcer with hemorrhage, without mention of obstruction | X | — |
| ICD-9 | 534.41 | Chronic or unspecified gastrojejunal ulcer, with hemorrhage, with obstruction | X | — |
| ICD-9 | 534.6 | Chronic or unspecified gastrojejunal ulcer with hemorrhage and perforation | X | — |
| ICD-9 | 534.60 | Chronic or unspecified gastrojejunal ulcer with hemorrhage and perforation, without mention of obstruction | X | — |
| ICD-9 | 534.61 | Chronic or unspecified gastrojejunal ulcer with hemorrhage and perforation, with obstruction | X | — |
| ICD-9 | 535.01 | Acute gastritis, with hemorrhage | X | X |
| ICD-9 | 535.11 | Atrophic gastritis, with hemorrhage | X | — |
| ICD-9 | 535.21 | Gastric mucosal hypertrophy, with hemorrhage | X | — |
| ICD-9 | 535.41 | Other specified gastritis, with hemorrhage | X | — |
| ICD-9 | 535.51 | Unspecified gastritis and gastroduodenitis, with hemorrhage | X | — |
| ICD-9 | 535.61 | Duodenitis, with hemorrhage | X | — |
| ICD-9 | 537.83 | Angiodysplasia of stomach and duodenum with hemorrhage | X | — |
| ICD-9 | 537.84 | Dieulafoy lesion (hemorrhagic) of stomach and duodenum | X | — |
| ICD-9 | 562.02 | Diverticulosis of small intestine with hemorrhage | X | — |
| ICD-9 | 562.03 | Diverticulitis of small intestine with hemorrhage | X | — |
| ICD-9 | 562.12 | Diverticulosis of colon with hemorrhage | X | — |
| ICD-9 | 562.13 | Diverticulitis of colon with hemorrhage | X | — |
| ICD-9 | 568.81 | Hemoperitoneum (nontraumatic) | X | X |
| ICD-9 | 569.3 | Hemorrhage of rectum and anus | X | — |
| ICD-9 | 569.85 | Angiodysplasia of intestine with hemorrhage | X | — |
| ICD-9 | 578 | Gastrointestinal hemorrhage | X | — |
| ICD-9 | 578.0 | Hematemesis | X | — |
| ICD-9 | 578.1 | Blood in stool | X | — |
| ICD-9 | 578.9 | Hemorrhage of gastrointestinal tract, unspecified | X | — |
| ICD-9 | 596.7 | Hemorrhage into bladder wall | X | — |
| ICD-9 | 599.70 | hematuria, unspecified | X | — |
| ICD-9 | 599.71 | Gross hematuria | X | — |
| ICD-9 | 620.7 | Hematoma of broad ligament | X | — |
| ICD-9 | 624.5 | Hematoma of vulva | X | — |
| ICD-9 | 626.6 | Metrorrhagia | X | — |
| ICD-9 | 626.8 | Other disorders of menstruation and other abnormal bleeding from female genital tract | X | — |
| ICD-9 | 626.9 | Unspecified disorders of menstruation and other abnormal bleeding from female genital tract | X | — |
| ICD-9 | 627.0 | Premenopausal menorrhagia | X | — |
| ICD-9 | 627.1 | postmenopausal bleeding | X | — |
| ICD-9 | 719.1 | Hemarthrosis | X | — |
| ICD-9 | 719.10 | hemarthrosis site unspecified | X | — |
| ICD-9 | 719.11 | Hemarthrosis, shoulder region | X | — |
| ICD-9 | 719.12 | Hemarthrosis, upper arm | X | — |
| ICD-9 | 719.13 | Hemarthrosis, forearm | X | — |
| ICD-9 | 719.14 | Hemarthrosis, hand | X | — |
| ICD-9 | 719.15 | Hemarthrosis, pelvic region and thigh | X | — |
| ICD-9 | 719.16 | Hemarthrosis, lower leg | X | — |
| ICD-9 | 719.17 | Hemarthrosis, ankle and foot | X | — |
| ICD-9 | 719.18 | Hemarthrosis, other specified sites | X | — |
| ICD-9 | 719.19 | Hemarthrosis, multiple sites | X | — |
| ICD-9 | 729.7 | Nontraumatic compartment syndrome | X | X |
| ICD-9 | 729.71 | Nontraumatic compartment syndrome of upper extremity | X | X |
| ICD-9 | 729.72 | Nontraumatic compartment syndrome of lower extremity | X | X |
| ICD-9 | 729.73 | Nontraumatic compartment syndrome of abdomen | X | X |
| ICD-9 | 729.79 | Nontraumatic compartment syndrome of other sites | X | X |
| ICD-9 | 729.92 | Nontraumatic hematoma of soft tissue | X | — |
| ICD-9 | 784.7 | Epistaxis | X | — |
| ICD-9 | 784.8 | bleeding from throat | X | — |
| ICD-9 | 786.30 | Hemoptysis, unspecified | X | — |
| ICD-9 | 786.39 | Other hemoptysis | X | — |
| ICD-9 | 852 | Subarachnoid subdural and extradural hemorrhage following injury | X | X |
| ICD-9 | 852.0 | Subarachnoid hemorrhage following injury without mention of open intracranial wound | X | X |
| ICD-9 | 852.00 | Subarachnoid hemorrhage following injury without mention of open intracranial wound, unspecified state of consciousness | X | X |
| ICD-9 | 852.01 | Subarachnoid hemorrhage following injury without mention of open intracranial wound, with no loss of consciousness | X | X |
| ICD-9 | 852.02 | Subarachnoid hemorrhage following injury without mention of open intracranial wound, with brief [less than one hour] loss of consciousness | X | X |
| ICD-9 | 852.03 | Subarachnoid hemorrhage following injury without mention of open intracranial wound, with moderate [1-24 hours] loss of consciousness | X | X |
| ICD-9 | 852.04 | Subarachnoid hemorrhage following injury without mention of open intracranial wound, with prolonged [more than 24 hours] loss of consciousness and return to pre-existing conscious level | X | X |
| ICD-9 | 852.05 | Subarachnoid hemorrhage following injury without mention of open intracranial wound, with prolonged [more than 24 hours] loss of consciousness without return to pre-existing conscious level | X | X |
| ICD-9 | 852.06 | Subarachnoid hemorrhage following injury without mention of open intracranial wound, with loss of consciousness of unspecified duration | X | X |
| ICD-9 | 852.09 | Subarachnoid hemorrhage following injury without mention of open intracranial wound, with concussion, unspecified | X | X |
| ICD-9 | 852.1 | Subarachnoid hemorrhage following injury with open intracranial wound | X | X |
| ICD-9 | 852.10 | Subarachnoid hemorrhage following injury with open intracranial wound, unspecified state of consciousness | X | X |
| ICD-9 | 852.11 | Subarachnoid hemorrhage following injury with open intracranial wound, with no loss of consciousness | X | X |
| ICD-9 | 852.12 | Subarachnoid hemorrhage following injury with open intracranial wound, with brief [less than one hour] loss of consciousness | X | X |
| ICD-9 | 852.13 | Subarachnoid hemorrhage following injury with open intracranial wound, with moderate [1-24 hours] loss of consciousness | X | X |
| ICD-9 | 852.14 | Subarachnoid hemorrhage following injury with open intracranial wound, with prolonged [more than 24 hours) loss of consciousness and return to pre-existing conscious level | X | X |
| ICD-9 | 852.15 | Subarachnoid hemorrhage following injury with open intracranial wound, with prolonged [more than 24 hours] loss of consciousness without return to pre-existing conscious level | X | X |
| ICD-9 | 852.16 | Subarachnoid hemorrhage following injury with open intracranial wound, with loss of consciousness of unspecified duration | X | X |
| ICD-9 | 852.19 | Subarachnoid hemorrhage following injury with open intracranial wound, with concussion, unspecified | X | X |
| ICD-9 | 852.2 | Subdural hemorrhage following injury without mention of open intracranial wound | X | X |
| ICD-9 | 852.20 | Subdural hemorrhage following injury without mention of open intracranial wound, unspecified state of consciousness | X | X |
| ICD-9 | 852.21 | Subdural hemorrhage following injury without mention of open intracranial wound, with no loss of consciousness | X | X |
| ICD-9 | 852.22 | Subdural hemorrhage following injury without mention of open intracranial wound, with brief [less than one hour] loss of consciousness | X | X |
| ICD-9 | 852.23 | Subdural hemorrhage following injury without mention of open intracranial wound, with moderate [1-24 hours] loss of consciousness | X | X |
| ICD-9 | 852.24 | Subdural hemorrhage following injury without mention of open intracranial wound, with prolonged [more than 24 hours] loss of consciousness and return to pre-existing conscious level | X | X |
| ICD-9 | 852.25 | Subdural hemorrhage following injury without mention of open intracranial wound, with prolonged [more than 24 hours] loss of consciousness without return to pre-existing conscious level | X | X |
| ICD-9 | 852.26 | Subdural hemorrhage following injury without mention of open intracranial wound, with loss of consciousness of unspecified duration | X | X |
| ICD-9 | 852.29 | Subdural hemorrhage following injury without mention of open intracranial wound, with concussion, unspecified | X | X |
| ICD-9 | 852.3 | Subdural hemorrhage following injury with open intracranial wound | X | X |
| ICD-9 | 852.30 | Subdural hemorrhage following injury with open intracranial wound, unspecified state of consciousness | X | X |
| ICD-9 | 852.31 | Subdural hemorrhage following injury with open intracranial wound, with no loss of consciousness | X | X |
| ICD-9 | 852.32 | Subdural hemorrhage following injury with open intracranial wound, with brief [less than one hour] loss of consciousness | X | X |
| ICD-9 | 852.33 | Subdural hemorrhage following injury with open intracranial wound, with moderate [1-24 hours] loss of consciousness | X | X |
| ICD-9 | 852.34 | Subdural hemorrhage following injury with open intracranial wound, with prolonged [more than 24 hours] loss of consciousness and return to pre-existing conscious level | X | X |
| ICD-9 | 852.35 | Subdural hemorrhage following injury with open intracranial wound, with prolonged [more than 24 hours] loss of consciousness without return to pre-existing conscious level | X | X |
| ICD-9 | 852.36 | Subdural hemorrhage following injury with open intracranial wound, with loss of consciousness of unspecified duration | X | X |
| ICD-9 | 852.39 | Subdural hemorrhage following injury with open intracranial wound, with concussion, unspecified | X | X |
| ICD-9 | 852.4 | Extradural hemorrhage following injury without mention of open intracranial wound | X | X |
| ICD-9 | 852.40 | Extradural hemorrhage following injury without mention of open intracranial wound, unspecified state of consciousness | X | X |
| ICD-9 | 852.41 | Extradural hemorrhage following injury without mention of open intracranial wound, with no loss of consciousness | X | X |
| ICD-9 | 852.42 | Extradural hemorrhage following injury without mention of open intracranial wound, with brief [less than 1 hour] loss of consciousness | X | X |
| ICD-9 | 852.43 | Extradural hemorrhage following injury without mention of open intracranial wound, with moderate [1-24 hours] loss of consciousness | X | X |
| ICD-9 | 852.44 | Extradural hemorrhage following injury without mention of open intracranial wound, with prolonged [more than 24 hours] loss of consciousness and return to pre-existing conscious level | X | X |
| ICD-9 | 852.45 | Extradural hemorrhage following injury without mention of open intracranial wound, with prolonged [more than 24 hours] loss of consciousness without return to pre-existing conscious level | X | X |
| ICD-9 | 852.46 | Extradural hemorrhage following injury without mention of open intracranial wound, with loss of consciousness of unspecified duration | X | X |
| ICD-9 | 852.49 | Extradural hemorrhage following injury without mention of open intracranial wound, with concussion, unspecified | X | X |
| ICD-9 | 852.5 | Extradural hemorrhage following injury with open intracranial wound | X | X |
| ICD-9 | 852.50 | Extradural hemorrhage following injury with open intracranial wound, unspecified state of consciousness | X | X |
| ICD-9 | 852.51 | Extradural hemorrhage following injury with open intracranial wound, with no loss of consciousness | X | X |
| ICD-9 | 852.52 | Extradural hemorrhage following injury with open intracranial wound, with brief [less than one hour] loss of consciousness | X | X |
| ICD-9 | 852.53 | Extradural hemorrhage following injury with open intracranial wound, with moderate [1-24 hours] loss of consciousness | X | X |
| ICD-9 | 852.54 | Extradural hemorrhage following injury with open intracranial wound, with prolonged [more than 24 hours] loss of consciousness and return to pre-existing conscious level | X | X |
| ICD-9 | 852.55 | Extradural hemorrhage following injury with open intracranial wound, with prolonged [more than 24 hours] loss of consciousness without return to pre-existing conscious level | X | X |
| ICD-9 | 852.56 | Extradural hemorrhage following injury with open intracranial wound, with loss of consciousness of unspecified duration | X | X |
| ICD-9 | 852.59 | Extradural hemorrhage following injury with open intracranial wound, with concussion, unspecified | X | X |
| ICD-9 | 853 | Other and unspecified intracranial hemorrhage following injury | X | X |
| ICD-9 | 853.0 | Other and unspecified intracranial hemorrhage following injury without mention of open intracranial wound | X | X |
| ICD-9 | 853.00 | Other and unspecified intracranial hemorrhage following injury without mention of open intracranial wound, unspecified state of consciousness | X | X |
| ICD-9 | 853.01 | Other and unspecified intracranial hemorrhage following injury without mention of open intracranial wound, with no loss of consciousness | X | X |
| ICD-9 | 853.02 | Other and unspecified intracranial hemorrhage following injury without mention of open intracranial wound, with brief [less than one hour] loss of consciousness | X | X |
| ICD-9 | 853.03 | Other and unspecified intracranial hemorrhage following injury without mention of open intracranial wound, with moderate [1-24 hours] loss of consciousness | X | X |
| ICD-9 | 853.04 | Other and unspecified intracranial hemorrhage following injury without mention of open intracranial wound, with prolonged [more than 24 hours] loss of consciousness and return to pre- existing conscious level | X | X |
| ICD-9 | 853.05 | Other and unspecified intracranial hemorrhage following injury without mention of open intracranial wound, with prolonged [more than 24 hours] loss of consciousness without return to pre-existing conscious level | X | X |
| ICD-9 | 853.06 | Other and unspecified intracranial hemorrhage following injury without mention of open intracranial wound, with loss of consciousness of unspecified duration | X | X |
| ICD-9 | 853.09 | Other and unspecified intracranial hemorrhage following injury without mention of open intracranial wound, with concussion, unspecified | X | X |
| ICD-9 | 853.1 | Other and unspecified intracranial hemorrhage following injury with open intracranial wound | X | X |
| ICD-9 | 853.10 | Other and unspecified intracranial hemorrhage following injury with open intracranial wound, unspecified state of consciousness | X | X |
| ICD-9 | 853.11 | Other and unspecified intracranial hemorrhage following injury with open intracranial wound, with no loss of consciousness | X | X |
| ICD-9 | 853.12 | Other and unspecified intracranial hemorrhage following injury with open intracranial wound, with brief [less than one hour] loss of consciousness | X | X |
| ICD-9 | 853.13 | Other and unspecified intracranial hemorrhage following injury with open intracranial wound, with moderate [1-24 hours] loss of consciousness | X | X |
| ICD-9 | 853.14 | Other and unspecified intracranial hemorrhage following injury with open intracranial wound, with prolonged [more than 24 hours] loss of consciousness and return to pre-existing conscious level | X | X |
| ICD-9 | 853.15 | Other and unspecified intracranial hemorrhage following injury with open intracranial wound, with prolonged [more than 24 hours] loss of consciousness without return to pre-existing conscious level | X | X |
| ICD-9 | 853.16 | Other and unspecified intracranial hemorrhage following injury with open intracranial wound, with loss of consciousness of unspecified duration | X | X |
| ICD-9 | 853.19 | Other and unspecified intracranial hemorrhage following injury with open intracranial wound, with concussion, unspecified | X | X |
| ICD-9 | 864.01 | Injury to liver without mention of open wound into cavity, hematoma and contusion | X | X |
| ICD-9 | 864.11 | Injury to liver with open wound into cavity, hematoma and contusion | X | X |
| ICD-9 | 865.01 | Injury to spleen without mention of open wound into cavity, hematoma without rupture of capsule | X | X |
| ICD-9 | 865.11 | Injury to spleen with open wound into cavity, hematoma without rupture of capsule | X | X |
| ICD-9 | 866.01 | Injury to kidney without mention of open wound into cavity, hematoma without rupture of capsule | X | X |
| ICD-9 | 866.11 | Injury to kidney with open wound into cavity, hematoma without rupture of capsule | X | X |
| ICD-9 | 958.9 | Traumatic compartment syndrome | X | X |
| ICD-9 | 958.90 | Compartment syndrome, unspecified | X | X |
| ICD-9 | 958.91 | Traumatic compartment syndrome of upper extremity | X | X |
| ICD-9 | 958.92 | Traumatic compartment syndrome of lower extremity | X | X |
| ICD-9 | 958.93 | Traumatic compartment syndrome of abdomen | X | X |
| ICD-9 | 958.99 | Traumatic compartment syndrome of other sites | X | X |

“X” represents codes that were included for identification of the endpoint under the specified setting. “—" represents codes that were reviewed with a decision to not include.

Note: * Must be accompanied by a hematoma or hemorrhage code during the same hospitalization

## **Other Supplement Tables and Figures**

**Table S4: Baseline characteristics for patients with acute medical illness**

|  | **Acute Illness** | | | | | | **TOTAL**  **N = 58,186** |
| --- | --- | --- | --- | --- | --- | --- | --- |
|  | **Infection,**  **N = 32,893** | **Respiratory**  **Insufficiency,**  **N = 17,918** | **Ischemic Stroke,**  **N = 4,881** | **Inflammatory Condition,**  **N = 4,774** | **Cancer,**  **N = 4,688** | **Heart Failure,**  **N = 4,596** |  |
| **Demographics** |  |  |  |  |  |  |  |
| **Female sex, N (%)** | 54.2 | 58.9 | 52.4 | 56.9 | 55.4 | 51.3 | 55.2 |
| **Age, years (%)** |  |  |  |  |  |  |  |
| 18–39 years | 14.3 | 10.2 | 4.2 | 9.3 | 6.1 | 7.1 | 11.6 |
| 40–64 years | 55.7 | 53.4 | 51.6 | 53.5 | 60.2 | 49.3 | 55.3 |
| 65–75 years | 18.5 | 22.8 | 26.0 | 23.3 | 23.5 | 24.0 | 20.6 |
| > 75 years | 11.5 | 13.6 | 18.1 | 13.9 | 10.1 | 19.6 | 12.5 |
| **BMI kg/m^2^ (%)** |  |  |  |  |  |  |  |
| 30–34.9 | 43.8 | 41.7 | 55.2 | 47.4 | 56.2 | 34.5 | 44.7 |
| 35–39.9 | 25.3 | 24.3 | 25.4 | 27.1 | 25.3 | 25.0 | 25.3 |
| > 40 | 30.9 | 33.9 | 19.4 | 25.5 | 18.6 | 40.6 | 29.9 |
| **Index hospitalization characteristics (at follow-up)** |  |  |  |  |  |  |  |
| Length of hospital stay (days), median (IQR) | 5 (4–8) | 5 (4–8) | 4 (3–7) | 4 (3–7) | 5 (3–8) | 5 (4–8) | 5 (3–7) |
| Admission to enoxaparin start (days), median (IQR) | 1 (0–1) | 0 (0–1) | 1 (0–1) | 1 (0–1) | 1 (0–1) | 0 (0–1) | 1 (0–1) |
| Enoxaparin start to discharge (days), median (IQR) | 4 (3–7) | 5 (3–7) | 3 (2–6) | 3 (2–6) | 4 (2–7) | 5 (3–7) | 4 (3–6) |
| Duration of prophylaxis with enoxaparin, median and IQR | 4 (2–6) | 4 (2–6) | 3 (2–5) | 3 (1–5) | 3 (1–6) | 4 (2–6) | 3 (2–5) |
| Patients with enoxaparin Rx post discharge (%) | 0.8 | 1.1 | 1.8 | 3.4 | 4.0 | 1.0 | 1.3 |
| **Stroke and CBVD (%)** |  |  |  |  |  |  |  |
| Hemorrhagic stroke | 0.3 | 0.3 | 0.7 | 0.2 | 0.9 | 0.3 | 0.4 |
| Ischemic stroke | 3.1 | 3.6 | 30.2 | 3.0 | 3.1 | 5.0 | 5.2 |
| Unspecified stroke or CBVD without stroke | 7.0 | 13.5 | 5.8 | 7.4 | 7.9 | 12.6 | 8.7 |
| **Other Comorbidities (%)** |  |  |  |  |  |  |  |
| Thrombophilia | 0.4 | 0.4 | 1.2 | 0.8 | 0.6 | 0.4 | 0.5 |
| Severe varicosities | 2.2 | 2.2 | 1.5 | 1.8 | 1.6 | 3.1 | 2.1 |
| History of cancer | 9.5 | 4.3 | 7.7 | 7.7 | 83.5 | 8.5 | 14.3 |
| Gastroduodenal ulcer | 4.8 | 5.7 | 3.4 | 9.1 | 7.3 | 4.3 | 5.0 |
| Lower limb paralysis | 0.8 | 0.5 | 0.5 | 0.8 | 0.6 | 0.4 | 0.7 |
| Central venous catheter | 4.4 | 4.3 | 1.5 | 3.4 | 15.7 | 4.2 | 4.6 |
| Heart failure | 8.0 | 15.8 | 6.2 | 6.5 | 5.0 | 41.9 | 10.9 |
| COPD | 16.7 | 39.4 | 11.4 | 11.0 | 13.4 | 30.6 | 21.0 |
| CKD stage III | 18.1 | 19.5 | 19.5 | 15.3 | 14.4 | 32.4 | 18.4 |
| CHD | 14.9 | 22.0 | 19.8 | 15.5 | 14.4 | 36.4 | 17.9 |
| Drug misuse disorder^a^ | 20.6 | 30.9 | 20.2 | 18.6 | 19.5 | 26.1 | 23.2 |
| HIV infection | 1.5 | 1.5 | 0.7 | 1.1 | 1.3 | 0.8 | 1.4 |
| Current or former of tobacco use | 43.7 | 56.7 | 40.6 | 45.7 | 53.8 | 51.7 | 47.5 |
| Hypertension | 50.8 | 57.4 | 59.0 | 55.6 | 56.4 | 66.6 | 54.3 |
| Diabetes | 34.4 | 35.4 | 36.9 | 27.8 | 27.2 | 47.1 | 34.2 |
| Anemia | 12.7 | 14.5 | 7.7 | 13.9 | 20.5 | 17.2 | 13.3 |
| Immuno-hematologic conditions^b^ | 1.7 | 1.6 | 0.7 | 1.3 | 10.5 | 1.4 | 2.0 |
| Peripheral vascular disease | 9.0 | 10.7 | 9.8 | 8.7 | 7.0 | 13.9 | 9.3 |
| Moderate/severe chronic liver disease | 6.3 | 6.9 | 3.9 | 5.5 | 8.9 | 6.0 | 6.3 |
| **History of VTE and Bleeding (%)** |  |  |  |  |  |  |  |
| History of VTE | 4.1 | 5.0 | 3.6 | 5.9 | 4.4 | 5.5 | 4.5 |
| History of bleeding |  |  |  |  |  |  |  |
| Major bleeding | 1.1 | 1.2 | 2.3 | 1.1 | 1.9 | 1.5 | 1.3 |
| Nonmajor bleeding | 10.1 | 10.1 | 6.5 | 9.3 | 17.3 | 8.9 | 10.0 |
| **Medication Use (%)** |  |  |  |  |  |  |  |
| Anticoagulants^c^ | 22.2 | 24.9 | 24.4 | 27.8 | 40.3 | 26.8 | 24.9 |
| ACEi/ ARB | 29.5 | 31.6 | 36.3 | 32.4 | 29.9 | 40.6 | 31.2 |
| Beta-blockers | 12.9 | 15.5 | 16.4 | 16.0 | 17.5 | 21.0 | 14.7 |
| CCBs | 14.2 | 18.8 | 21.8 | 15.4 | 15.6 | 18.6 | 16.0 |
| Statins | 24.2 | 27.6 | 35.6 | 26.9 | 23.3 | 31.9 | 26.1 |
| Antiplatelets^c^ | 2.7 | 4.2 | 13.9 | 2.5 | 1.7 | 7.2 | 4.1 |
| Hormone replacement therapy | 0.9 | 0.6 | 0.4 | 0.8 | 0.5 | 0.3 | 0.7 |

Abbreviations: ACEi, angiotensin-converting enzyme inhibitors; ARB, angiotensin II receptor blockers; CBVD, cerebrovascular disease; CCBs, calcium channel blockers; CHD, coronary heart disease; CKD, chronic kidney disease; COPD, chronic obstructive pulmonary disease; HIV, human immunodeficiency virus; HR, hazard ratio; PAD, peripheral artery disease; VTE, venous thromboembolism.

* Stratification by illness type is purely descriptive, and all inferential analyses will treat the cohort as a single population with illness type as a covariate

a Includes codes for nicotine dependence, opioid dependence, other psychoactive substance abuse, other illegal prescription drug abuse. b Includes anemia, cell aplasia, pancytopenia, bone marrow failure syndromes, agranulocytosis, genetic anomalies of leukocytes, severe combined immunodeficiency (SCID), Nezelof's syndrome, Wiskott-Aldrich syndrome, Di George's syndrome, acute graft-versus-host disease. c Within 30-90 days of index.

**Table S5:** **Enoxaparin prophylaxis dosage by cause of hospitalization**

| **Reasons for hospitalization** | **Enoxaparin dosage (mg)**  **Mean (SD)** |
| --- | --- |
| Acute Infections | 65.4 (40.5) |
| Respiratory Insufficiency | 68.3 (46.5) |
| Ischemic Stroke | 64.9 (37.1) |
| Inflammatory Diseases | 71.8 (49.8) |
| Cancer | 71.4 (53.7) |
| Heart Failure | 73.3 (52.6) |

**Table S6: Event Rates for VTE and Major Bleeding in-hospital vs post-discharge**

|  |  | **VTE at 90 days follow up** | | | **Major Bleeding at 90 days follow up** | | |
| --- | --- | --- | --- | --- | --- | --- | --- |
|  | **N (%)** | **Overall Event Rate (%)** | **Events in-hospital (%)** | **Events post-discharge**  **(%)** | **Overall Event Rate (%)** | **Events in-hospital (%)** | **Events post-discharge**  **(%)** |
| Acute illness overall | 58,186 (65.6) | 3.2 | 0.7 | 2.5 | 1.8 | 0.6 | 1.2 |
| Infection | 32,893 (56.5) | 2.8 | 0.8 | 2.0 | 1.6 | 0.6 | 1.0 |
| Respiratory Insufficiency | 17,918 (30.8) | 3.7 | 1.0 | 2.7 | 2.1 | 0.9 | 1.2 |
| Cancer | 4,688 (8.1) | 7.8 | 1.1 | 6.7 | 3.0 | 0.8 | 2.2 |
| Ischemic Stroke | 4,881 (8.3) | 3.3 | 0.9 | 2.4 | 3.7 | 1.6 | 2.1 |
| Inflammatory Condition | 4,774 (8.2) | 3.4 | 0.7 | 2.7 | 1.5 | 0.7 | 0.8 |
| Heart Failure | 4,596 (7.9) | 3.6 | 0.8 | 2.8 | 2.8 | 1.0 | 1.8 |

Abbreviations: VTE, venous thromboembolism.

Event rates represent the 1 – Kaplan Meier estimates.

**Figure S1. Enoxaparin prophylaxis dosage from index for acute illness**


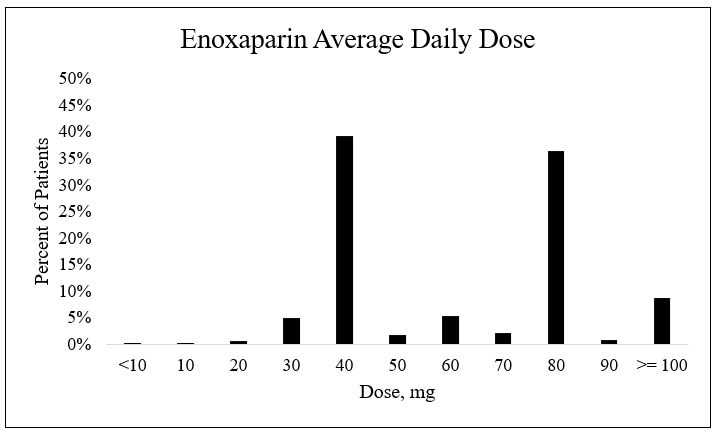


**Figure S2. VTE and Major Bleeding Event Rates for Usual Dose vs High Dose**

**
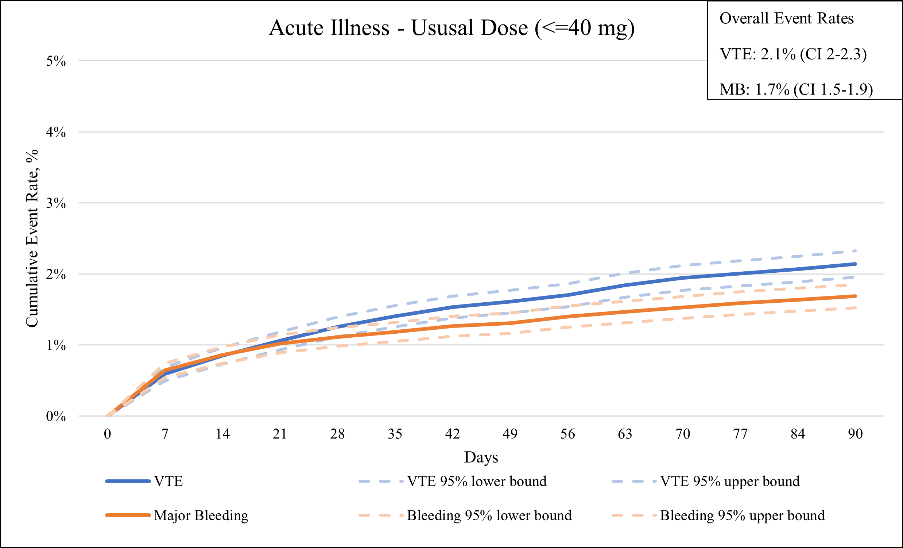
**

**
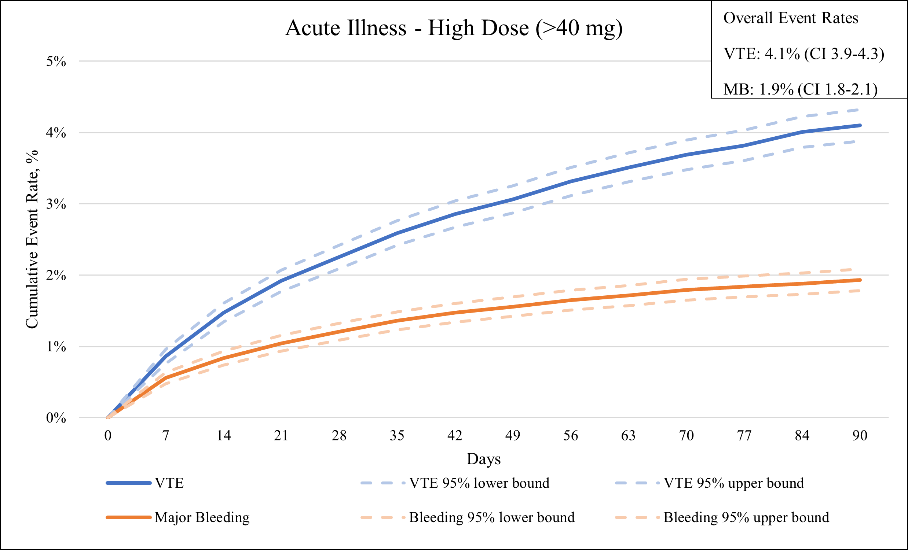
**

Time zero indicates enoxaparin initiation. KM, Kaplan-Meier; and VTE, venous thromboembolism

## **Supplement References**

[1] Dawwas GK, Leonard CE, Lewis JD, Cuker A. Risk for Recurrent Venous Thromboembolism and Bleeding With Apixaban Compared With Rivaroxaban: An Analysis of Real-World Data. Ann Intern Med. 2022;175:20-8. doi: 10.7326/M21-0717.

[2] Pawar A, Gagne JJ, Gopalakrishnan C, Iyer G, Tesfaye H, Brill G et al. Association of Type of Oral Anticoagulant Dispensed With Adverse Clinical Outcomes in Patients Extending Anticoagulation Therapy Beyond 90 Days After Hospitalization for Venous Thromboembolism. JAMA. 2022;327:1051-60. doi: 10.1001/jama.2022.1920.

[3] Albertsen IE, Nielsen PB, Søgaard M, Goldhaber SZ, Overvad TF, Rasmussen LH et al. Risk of Recurrent Venous Thromboembolism: A Danish Nationwide Cohort Study. Am J Med. 2018;131:1067-74.e4. doi: 10.1016/j.amjmed.2018.04.042.

[4] Larsen TB, Skjøth F, Kjældgaard JN, Lip GY, Nielsen PB, Søgaard M. Effectiveness and safety of rivaroxaban and warfarin in patients with unprovoked venous thromboembolism: a propensity-matched nationwide cohort study. Lancet Haematol. 2017; 4:e237-44. doi:10.1016/S2352-3026(17)30054-6.

[5] White RH, Garcia M, Sadeghi B, Tancredi DJ, Zrelak P, Cuny J et al. Evaluation of the predictive value of ICD-9-CM coded administrative data for venous thromboembolism in the United States. Thromb Res. 2010;126:61-7. doi: 10.1016/j.thromres.2010.03.009.

[6] Sundbøll J, Adelborg K, Munch T, Frøslev T, Sørensen HT, Bøtker HE et al. Positive predictive value of cardiovascular diagnoses in the Danish National Patient Registry: a validation study. BMJ Open. 2016 Nov 18;6(11):e012832. doi: 10.1136/bmjopen-2016-012832..

[7] Schulman S, Kearon C. Subcommittee on Control of Anticoagulation of the Scientific and Standardization Committee of the International Society on Thrombosis and Haemostasis. Definition of major bleeding in clinical investigations of antihemostatic medicinal products in non-surgical patients. J Thromb haemost. 2005 Apr 4;3(4):692-4. doi:10.1111/j.1538-7836.2005.01204.x

[8] Navar AM, Peterson ED, Steen DL, Wojdyla DM, Sanchez RJ, Khan I et al. Evaluation of Mortality Data From the Social Security Administration Death Master File for Clinical Research. JAMA Cardiol. 2019;4:375-9. doi: 10.1001/jamacardio.2019.0198.

[9] Cunningham A, Stein CM, Chung CP, Daugherty JR, Smalley WE, Ray WA. An automated database case definition for serious bleeding related to oral anticoagulant use. Pharmacoepidemiol Drug Saf. 2011;20:560-6. doi: 10.1002/pds.2109.

[10] Shehab N, Ziemba R, Campbell KN, Geller AI, Moro RN, Gage BF et al. Assessment of ICD-10-CM code assignment validity for case finding of outpatient anticoagulant-related bleeding among Medicare beneficiaries. Pharmacoepidemiol Drug Saf. 2019;28:951-964. doi: 10.1002/pds.4783.
